# Tracking total spending on tuberculosis by source and function in 135 low-income and middle-income countries, 2000–17: a financial modelling study

**DOI:** 10.1016/S1473-3099(20)30124-9

**Published:** 2020-08

**Authors:** Yanfang Su, Ines Garcia Baena, Anton C Harle, Sawyer W Crosby, Angela E Micah, Andrew Siroka, Maitreyi Sahu, Golsum Tsakalos, Christopher J L Murray, Katherine Floyd, Joseph L Dieleman

**Affiliations:** a Institute for Health Metrics and Evaluation, Seattle, WA, USA; b World Health Organization, Geneva, Switzerland

## Abstract

**Background:**

Estimates of government spending and development assistance for tuberculosis exist, but less is known about out-of-pocket and prepaid private spending. We aimed to provide comprehensive estimates of total spending on tuberculosis in low-income and middle-income countries for 2000–17.

**Methods:**

We extracted data on tuberculosis spending, unit costs, and health-care use from the WHO global tuberculosis database, Global Fund proposals and reports, National Health Accounts, the WHO-Choosing Interventions that are Cost-Effective project database, and the Institute for Health Metrics and Evaluation Development Assistance for Health Database. We extracted data from at least one of these sources for all 135 low-income and middle-income countries using the World Bank 2019 definitions. We estimated tuberculosis spending by source and function for notified (officially reported) and non-notified tuberculosis cases separately and combined, using spatiotemporal Gaussian process regression to fill in for missing data and estimate uncertainty. We aggregated estimates of government, out-of-pocket, prepaid private, and development assistance spending on tuberculosis to estimate total spending in 2019 US$.

**Findings:**

Total spending on tuberculosis in 135 low-income and middle-income countries increased annually by 3·9% (95% CI 3·0 to 4·6), from $5·7 billion (5·2 to 6·5) in 2000 to $10·9 billion (10·3 to 11·8) in 2017. Government spending increased annually by 5·1% (4·4 to 5·7) between 2000 and 2017, and reached $6·9 billion (6·5 to 7·5) or 63·5% (59·2 to 66·8) of all tuberculosis spending in 2017. Of government spending, $5·8 billion (5·6 to 6·1) was spent on notified cases. Out-of-pocket spending decreased annually by 0·8% (−2·9 to 1·3), from $2·4 billion (1·9 to 3·1) in 2000 to $2·1 billion (1·6 to 2·7) in 2017. Development assistance for country-specific spending on tuberculosis increased from $54·6 million in 2000 to $1·1 billion in 2017. Administrative costs and development assistance for global projects related to tuberculosis care increased from $85·3 million in 2000 to $576·2 million in 2017. 30 high tuberculosis burden countries of low and middle income accounted for 73·7% (71·8–75·8) of tuberculosis spending in 2017.

**Interpretation:**

Despite substantial increases since 2000, funding for tuberculosis is still far short of global financing targets and out-of-pocket spending remains high in resource-constrained countries, posing a barrier to patient's access to care and treatment adherence. Of the 30 countries with a high-burden of tuberculosis, just over half were primarily funded by government, while others, especially lower-middle-income and low-income countries, were still primarily dependent on development assistance for tuberculosis or out-of-pocket health spending.

**Funding:**

Bill & Melinda Gates Foundation.

## Introduction

Despite being preventable and curable, tuberculosis accounted for up to 1·4 million deaths in low-income and middle-income countries in 2017.^1^, ^2^ In 2014, the World Health Assembly adopted the WHO End Tuberculosis Strategy, which set ambitious targets to reduce tuberculosis deaths by 95% and tuberculosis incidence by 90% compared with 2015 levels by 2035.^3^ The Stop TB Partnership published a 5-year investment plan (ie, The Global Plan) to end tuberculosis, which provided a roadmap for accelerating impact on the tuberculosis epidemic and reaching the first milestones of the WHO End Tuberculosis Strategy between 2016 and 2020. In 2018, at the first ever UN high-level meeting on tuberculosis, global financing targets agreed on by all member states were set for the first time.^4^ The targets are to mobilise at least US$13 billion per year by 2022 for spending on tuberculosis prevention, diagnosis, and treatment by national governments (including funding provided as international development assistance for health [DAH]) and an additional $2 billion per year for tuberculosis research in the 5 years from 2018 to 2022.

Estimates of spending on tuberculosis by governments in low-income and middle-income countries^2^, ^5^ and development assistance for tuberculosis^2^, ^5^, ^6^ are available. However, little is known about worldwide out-of-pocket and prepaid private spending on tuberculosis, despite increasing emphasis on the importance of expanding access to health insurance and making progress towards universal health coverage to ensure access to tuberculosis prevention, diagnosis, and treatment. The most recent data on spending by tuberculosis patients and their households are from national surveys^7^ and other country-specific studies.^8^

**Research in context**

**Evidence before this study**

We searched PubMed and Google on June 17, 2019, for publications since 2000 relating to tuberculosis spending, using the terms (“tuberculosis” AND (“financing” OR “spending”)). We identified 44 papers. We also searched PubMed and Google on July 15, and Nov 21, 2019, for publications since 2000 relating to patient costs for tuberculosis treatment using the terms (“tuberculosis” AND (“patient” OR “treatment” OR “medical”) AND “cost”). We identified 53 papers, although none provided systematic tracking of government, household, and donor spending on prevention and treatment of tuberculosis across a large set of countries. WHO has monitored government and international donor financing for tuberculosis and published results in the annual WHO Global Tuberculosis Report since 2002. The Institute for Health Metrics and Evaluation has estimated development assistance for tuberculosis for the period 1990–2017, and the latest data on government spending and development assistance for tuberculosis published by WHO are for the period 2006–18. WHO estimates of government and development assistance spending have focused on notified (officially reported) tuberculosis cases, and they have not included estimates of spending on non-notified cases. Household Health Expenditure and Utilisation Surveys, Demographic and Health Surveys, and National Tuberculosis Prevalence Surveys provide information about care seeking. Additionally, 14 national surveys of costs for patients with tuberculosis and their households, completed since 2015, provide some of the best nationally representative data on costs for tuberculosis-affected households, including direct out-of-pocket spending on medical care. However, no database containing comparable estimates of prepaid private or out-of-pocket spending on tuberculosis in low-income and middle-income countries exists.

**Added value of this study**

Our study adds value in five main ways. First, we extracted all available data on tuberculosis spending from multiple published and unpublished sources. Second, we applied spatiotemporal regression methods designed for the Global Burden of Diseases, Injuries, and Risk Factors study to identify outlying data, fill in missing data, and make comprehensive and comparable estimates with confidence intervals. Third, we provide the first comprehensive and comparable modelled estimates of out-of-pocket spending on tuberculosis in low-income and middle-income countries, highlighting the proportion of tuberculosis spending from out-of-pocket payments, as a reflection of the financial burden of tuberculosis on households. Fourth, we disaggregated spending by function, inclusive of the national tuberculosis programme, outpatient visits (pre-diagnosis and during treatment), inpatient admissions, and private drugs. We also estimated spending for both notified and non-notified cases, including spending that was from government, out-of-pocket, and prepaid private sources. Finally, we provide the first estimates of total spending on tuberculosis (from all sources, and for both notified and non-notified cases) in low-income and middle-income countries from 2000 to 2017, making comparisons across time and countries possible.

**Implications of all the available evidence**

The financial estimates across countries, time, and disease burden offer a holistic perspective to gain insights into resources invested in tuberculosis, enabling stakeholders to assess past investments, reforms, and policies, and develop informed plans for future investments. Insights from our estimations highlight the gap between actual spending and global financing targets.

We aimed to fill current knowledge gaps by estimating total spending on tuberculosis in low-income and middle-income countries for the period 2000–17 from all available sources, including government, DAH, out-of-pocket spending, and prepaid private payments.

## Methods

### Overview

To generate comprehensive estimates of total spending on tuberculosis in 135 low-income and middle-income countries, as defined by the 2019 definitions of low-income and middle-income countries published by the World Bank,^9^ we first estimated domestic spending on tuberculosis for each low-income and middle-income country from 2000 until 2017. Next, we combined these domestic spending estimates with existing estimates of development assistance for tuberculosis to produce estimates of total spending. For domestic funding, we included spending from all sources: the government, prepaid private, and out-of-pocket. For out-of-pocket spending, we restricted analysis to modelled direct medical spending (as opposed to direct non-medical spending—eg, transport spending or indirect economic costs due to tuberculosis, such as loss of income), for consistency with National Health Accounts. In addition to our country-level analysis, we aggregated countries by World Bank income group,^9^ Global Burden of Diseases, Injuries, and Risk Factors study (GBD) super-region,^10^ and high tuberculosis burden country group (as defined by WHO for the period 2016–20)^2^ to support priority setting and collective global action (appendix pp 2–5).^10^ In country-level presentations, we included the GBD high-income super-region country Argentina, because it was defined as an upper-middle-income country by The World Bank Group. However, at the aggregated level, we only presented six GBD super-regions, excluding the GBD high-income super-region. Additionally, we highlight high tuberculosis burden countries in our study. We present these data for a subset of 30 high tuberculosis burden countries, and India and China are high-burden countries of particular interest because among the top 30 countries they comprise at least 40% of all incident cases.^1^, ^2^

### Domestic spending

We estimated domestic spending on tuberculosis by financing source and function. The three financing sources for domestic spending were government, out-of-pocket, and prepaid private spending for tuberculosis. The four spending categories (health functions) were the national tuberculosis programme, outpatient care, inpatient care, and drugs other than those purchased by the national tuberculosis programme (ie, private drugs). We included spending on officially notified cases of tuberculosis (ie, those reported to national authorities and the national tuberculosis programme) and people with tuberculosis who were not officially notified.

We extracted and compiled data on domestic spending on tuberculosis from the WHO global tuberculosis database; national tuberculosis reports; proposals, concept notes, and funding landscaping documents for tuberculosis programmes from the Global Fund to Fight AIDS, Tuberculosis and Malaria; the National Health Accounts; and the Institute for Health Metrics and Evaluation (IHME) Development Assistance for Health Database. All spending data and associated data sources we used are in the appendix (pp 6–8). Tabulated data on estimates of tuberculosis spending from all sources for individual countries were scarce. Although tabulated data on government spending were relatively complete, tabulated data on out-of-pocket and prepaid private spending were sparse (appendix p 8).

We used data from the national tuberculosis programmes reported to WHO and estimated spending on outpatient and inpatient care using country-specific service use and country-specific unit cost data. Domestic curative spending was broken down into spending for notified tuberculosis cases and non-notified tuberculosis cases. For notified and non-notified cases, we estimated pre-diagnosis outpatient spending, diagnostic spending, drug spending, outpatient spending, and inpatient spending; all components are in table 1. For each of the components in table 1 that were not assumed to be funded by the national tuberculosis programme, we calculated estimates by taking the product of the estimated unit cost (ie, price per visit or bed day) and use estimates (ie, the number of outpatient visits or inpatient days). Each part of this estimation was done separately for cases of tuberculosis that were drug susceptible and multidrug resistant. Not all people with tuberculosis are treated by care providers that are linked and reported to the national tuberculosis programme. We assumed all non-notified cases (ie, the number of incident cases net the number of notified cases) were treated. On the basis of discussions with global experts on tuberculosis, we assumed that the share of spending on these non-notified cases for pre-diagnosis visits, drugs, outpatient visits during treatment, and inpatient care during treatment paid for by the government, private insurance, and out-of-pocket was the same as the overall share of these sources in National Health Accounts data.

**Table 1:** Tuberculosis domestic spending: key assumptions of financing source, variables, and data sources for notified and non-notified tuberculosis cases

	**Assumptions of financing source**	**Variables (data source)**
**Notified cases**		
Pre-diagnosis outpatient spending	Sourced in the same way as average patients in health systems	Notified cases (WHO Global Tuberculosis database); outpatient unit cost per visit (WHO-CHOICE and National Health Accounts); pre-diagnosis visits per case (Nhung et al,^11^ Pedrazzoli et al,^12^ and unpublished data from National Tuberculosis Patient Cost Surveys that have been compiled by the WHO Global Tuberculosis Programme); and outpatient spending from three sources—ie, government, out-of-pocket, and prepaid private insurance (National Health Accounts)
Diagnostic spending	Covered by national tuberculosis programme spending	National tuberculosis programme spending (national tuberculosis programme data)
Drug spending	Covered by national tuberculosis programme spending	National tuberculosis programme spending (national tuberculosis programme data)
Spending on outpatient treatment	Fully sourced by government	Notified cases (WHO Global Tuberculosis database); outpatient unit cost per visit (WHO-CHOICE and NHA); visits per case in treatment (WHO Global Tuberculosis database)
Spending on inpatient treatment	Fully sourced by government	Notified cases (WHO Global Tuberculosis database); inpatient unit cost per day (WHO-CHOICE and National Health Accounts); inpatient cases per notified cases (WHO Global Tuberculosis database); days per inpatient case in treatment (WHO Global Tuberculosis database)
**Non-notified cases**		
Pre-diagnosis outpatient spending	Sourced in the same way as average patients in health systems	Incident cases (IHME); notified cases (WHO Global Tuberculosis database); outpatient unit cost per visit (WHO-CHOICE and National Health Accounts); pre-diagnosis visits per case (Nhung et al,^11^ Pedrazzoli et al,^12^ and unpublished data from National Tuberculosis Patient Cost Surveys that have been compiled by the WHO Global Tuberculosis Programme); and outpatient spending from three sources—ie, government, out-of-pocket, and prepaid private insurance (National Health Accounts)
Diagnostic spending	$0* (because they do not access regulated diagnostics)	NA
Drug spending	Sourced by out-of-pocket and prepaid private spending	Incident cases (IHME); notified cases (WHO Global Tuberculosis database); drug unit cost per notified case (WHO Global Tuberculosis database); and drug spending from two sources—ie, out-of-pocket and prepaid private insurance (IHME)
Spending on outpatient treatment	Sourced in the same way as average patients in health systems	Incident cases (IHME); notified cases (WHO Global Tuberculosis database); outpatient unit cost per visit (WHO-CHOICE and National Health Accounts); visits per case in treatment (WHO Global Tuberculosis database); and outpatient spending from three sources—ie, government, out-of-pocket, and prepaid private insurance (IHME)
Spending on inpatient treatment	Sourced in the same way as average patients in health systems	Incident cases (IHME); notified cases (WHO Global Tuberculosis database); inpatient unit cost per day (WHO-CHOICE and National Health Accounts); inpatient cases per notified cases (WHO Global Tuberculosis database); days per inpatient case in treatment (WHO Global Tuberculosis database); and inpatient spending from three sources—ie, government, out-of-pocket and prepaid private insurance (National Health Accounts)

Notified cases were reported by the ministry of health from each country to WHO. Non-notified cases were estimated by incidence net notification. CHOICE=Choosing Interventions that are Cost-Effective project database. IHME=Institute for Health Metrics and Evaluation.

* Inflation-adjusted 2019 US$.

The unit costs of an outpatient visit (either before diagnosis or during treatment) and an inpatient bed-day during treatment were taken from the WHO Choosing Interventions that are Cost-Effective project for 2017 and were modelled for all years using unit costs from Moses et al.^13^ Data on the number of pre-diagnosis outpatient visits were extracted from Nhung et al,^11^ Pedrazzoli et al,^12^ and unpublished data from national tuberculosis patient cost surveys that have been compiled by the WHO Global Tuberculosis Programme. We extracted data on drug prices per patient and use of health services during treatment by notified patients with tuberculosis (ie, the number of days spent in hospital and the number of tuberculosis outpatient visits) from the WHO global tuberculosis database. We also extracted data on use of health services during treatment by notified patients with tuberculosis (ie, the number of days spent in hospital and the number of tuberculosis outpatient visits) from the WHO global tuberculosis database.

### Development assistance and total spending

After estimating domestic curative spending, we added estimates of development assistance for tuberculosis to yield total health spending.

We extracted data on development assistance for tuberculosis from the IHME Development Assistance for Health database in the Global Health Data Exchange website.^6^ We included data from all major international contributors to the fight against tuberculosis. Development assistance for tuberculosis consists of spending on country projects and spending on administration and global initiatives. Spending on research and development by development agencies was categorised as global initiatives. We assumed that spending for country projects was solely distributed through national tuberculosis programmes for purposes such as diagnosis, drugs, and patient support, without including spending on outpatient or inpatient services provided in the general health system. For administration and global initiatives, amounts were not disaggregated by country because this spending funds global initiatives rather than resources for a single country.

### Statistical analysis

We present total tuberculosis spending estimates in inflation-adjusted 2019 US$, in purchasing-power parity-adjusted $, and as a proportion of total health sector spending with 95% CIs (more details on currency conversions are in the appendix [p 27]). Government, out-of-pocket, DAH, and prepaid private spending on tuberculosis are reported as a proportion of total tuberculosis spending. National tuberculosis programme spending, outpatient spending, inpatient spending, and private drug spending are also reported as a proportion of total tuberculosis spending.

We estimated total tuberculosis spending per incident case and its growth rate with 95% CIs over time. We calculated estimates of tuberculosis spending per incident case by country group by aggregating spending by each group and dividing by the estimated number of incident cases across the entire group. We used this approach to ensure that the aggregated estimate represents the population in the group, rather than the governments in the group, and conforms to processes used in broader Financing Global Health and Global Burden of Disease research. These estimates represent the grouping as a whole, rather than an average of the countries.

We did sensitivity analyses to test our assumptions about the treatment rate of non-notified cases, health-care use by non-notified cases, and estimated incidence of tuberculosis (appendix p 28). We also estimated total spending per incident case by gross domestic product (GDP) per capita for 2000–17 and highlighted several countries of interest in comparisons.

For all modelling, we used spatiotemporal Gaussian process regression (ST-GPR) methods to ensure a complete and comparable time series and to estimate uncertainty for all model inputs.^14^ For national tuberculosis programme spending, we used ST-GPR estimates to fill in missing data. For all estimates of service use and unit costs, we used the modelled estimates. Further technical details about estimates of tuberculosis domestic spending are in the appendix (pp 12–24).

We did all analyses in R (version 3.6.0).

### Role of the funding source

The funder of this study had no role in study design, data collection, data analysis, data interpretation, or writing of the report. All authors had full access to all the data in the study, and the corresponding author had final responsibility for the decision to submit for publication.

## Results

**Figure 1:**
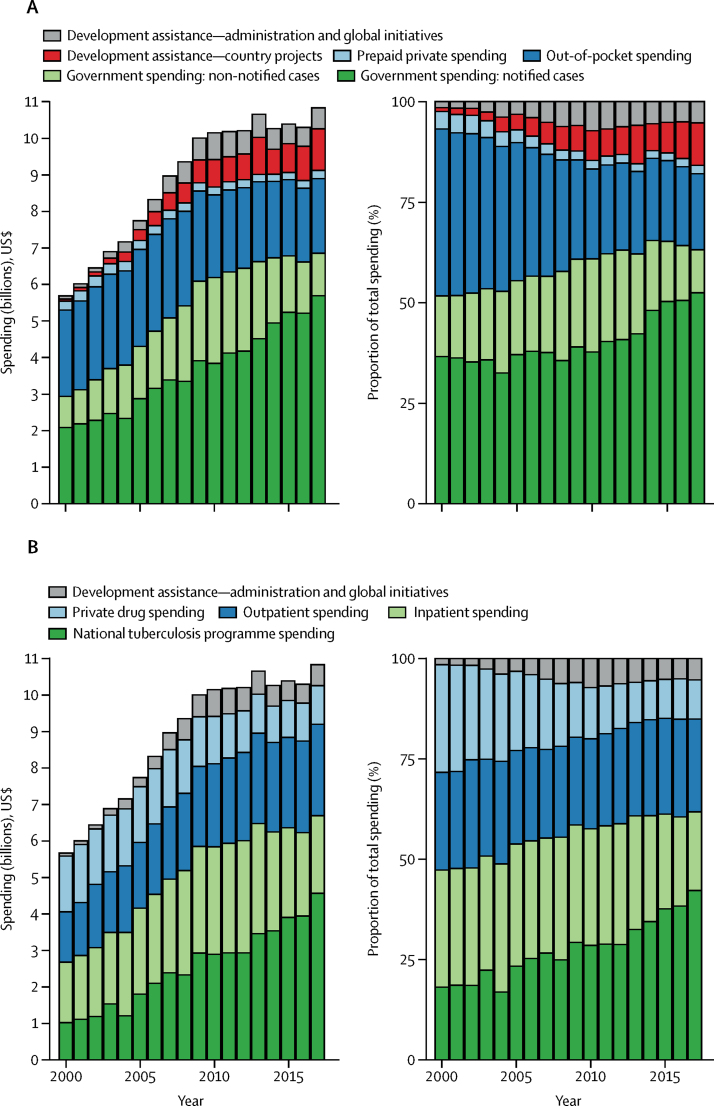
Tuberculosis spending by source (A) and function (B) in all low-income and middle-income countries, 2000–17 Spending is estimated for both notified and non-notified cases and presented in inflation-adjusted 2019 US$ and percentage.

Total spending on tuberculosis in low-income and middle-income countries increased by 3·9% per year (95% CI 3·0–4·6) between 2000 and 2017 (table 2), from $5·7 billion (5·2–6·5) in 2000 to $10·9 billion (10·3–11·8) in 2017 (figure 1).

**Table 2:** Tuberculosis spending in 135 low-income and middle-income countries, 2017

		**Total tuberculosis spending (millions), 2017, $**	**Total tuberculosis spending per incident case, 2017, $**	**DAH for tuberculosis as a fraction of total tuberculosis spending, 2017**	**Government tuberculosis spending as a fraction of total tuberculosis spending, 2017**	**Out-of-pocket tuberculosis spending as a fraction of total tuberculosis spending, 2017**	**Annual growth rate of total tuberculosis spending, 2000–17**
135 low-income and middle-income countries		10 941·9 (10 273·7 to 11 753·9)	1075·8 (1010·1 to 1155·7)	15·8% (14·7 to 16·8)	63·5% (59·2 to 66·8)	18·7% (15·2 to 23·6)	3·9% (3·0 to 4·6)
Aggregate categories							
	30 high-burden countries	7646·4 (7010·9 to 8457·9)	903·4 (828·3 to 999·2)	11·0% (9·9 to 12·0)	63·7% (57·7 to 68·4)	22·9% (17·9 to 29·6)	3·5% (2·4 to 4·6)
	India and China*	2931·6 (2382·1 to 3644·2)	778·5 (632·6 to 967·7)	3·9% (3·1 to 4·8)	59·0% (46·7 to 69·3)	34·3% (23·6 to 47·6)	2·8% (0·3 to 5·0)
	28 other high-burden countries	4714·9 (4356·0 to 5093·7)	1003·4 (927·1 to 1084·1)	15·4% (14·3 to 16·7)	66·6% (62·8 to 69·8)	15·7% (12·5 to 20·0)	4·1% (3·3 to 4·8)
	Other low-income and middle-income countries	2719·2 (2586·4 to 2861·8)	1593·7 (1515·9 to 1677·3)	11·3% (10·7 to 11·8)	76·5% (74·9 to 78·0)	10·9% (9·7 to 12·2)	3·9% (3·5 to 4·4)
World Bank income groups							
	Low-income countries	872·0 (833·9 to 913·3)	410·8 (392·9 to 430·3)	30·5% (29·1 to 31·8)	33·8% (31·7 to 35·8)	30·9% (28·5 to 33·7)	4·3% (3·9 to 4·8)
	Lower-middle-income countries	4060·4 (3595·6 to 4684·1)	654·6 (579·7 to 755·2)	17·2% (14·8 to 19·3)	47·8% (40·7 to 53·9)	32·5% (25·0 to 42·0)	5·7% (4·6 to 6·9)
	Upper-middle-income countries	5433·3 (4977·0 to 5952·6)	2944·2 (2697·0 to 3225·6)	3·4% (3·1 to 3·7)	86·7% (83·0 to 89·6)	8·3% (5·5 to 12·1)	2·4% (1·1 to 3·5)
GBD super-regions							
	Central Europe, eastern Europe, and central Asia	3370·8 (3057·8 to 3705·6)	13 954·7 (12 659·3 to 15 340·8)	3·1% (2·8 to 3·4)	88·6% (84·1 to 91·6)	7·8% (4·7 to 12·4)	4·8% (3·8 to 5·8)
	Latin America and Caribbean	518·3 (458·9 to 581·8)	2613·8 (2314·4 to 2934·2)	4·3% (3·8 to 4·9)	91·5% (89·7 to 92·9)	3·5% (2·4 to 5·1)	2·6% (1·6 to 3·7)
	North Africa and Middle East	326·7 (289·5 to 372·6)	1345·6 (1192·5 to 1534·8)	8·3% (7·2 to 9·3)	82·3% (79·1 to 85·2)	8·9% (6·6 to 11·8)	4·3% (3·1 to 5·5)
	South Asia	2166·7 (1720·8 to 2769·2)	615·8 (489·1 to 787·1)	10·6% (8·1 to 13·1)	44·1% (32·3 to 54·8)	42·8% (30·0 to 56·8)	6·3% (4·1 to 8·6)
	Southeast Asia, east Asia, and Oceania	1879·2 (1580·0 to 2257·2)	896·1 (753·5 to 1076·4)	9·9% (8·2 to 11·7)	74·7% (66·9 to 81·0)	13·4% (8·0 to 21·0)	0·4% (−1·9 to 2·6)
	Sub-Saharan Africa	2031·5 (1902·4 to 2196·4)	525·9 (492·5 to 568·6)	28·8% (26·6 to 30·7)	39·0% (35·4 to 42·7)	26·9% (23·2 to 32·0)	4·0% (3·3 to 4·7)
High tuberculosis burden low-income and middle-income countries							
	Angola	95·7 (80·8 to 114·9)	944·1 (797·5 to 1134·3)	9·4% (7·7 to 11·0)	60·0% (49·5 to 69·0)	25·6% (16·2 to 37·0)	9·4% (7·3 to 11·5)
	Bangladesh	89·2 (76·6 to 106·0)	460·4 (395·7 to 547·3)	53·9% (45·1 to 62·3)	25·2% (19·2 to 32·2)	20·5% (10·9 to 32·4)	6·7% (4·2 to 9·3)
	Brazil	95·9 (66·6 to 136·4)	1166·9 (811·0 to 1660·4)	0·1% (0·0 to 0·1)	91·5% (83·8 to 96·3)	7·2% (2·8 to 14·2)	0·7% (−2·4 to 3·7)
	Cambodia	43·0 (38·4 to 48·4)	1102·7 (986·7 to 1241·0)	28·9% (25·6 to 32·2)	67·8% (63·8 to 71·4)	3·3% (1·3 to 6·3)	3·8% (2·8 to 4·8)
	Central African Republic	12·7 (11·9 to 13·7)	366·0 (343·6 to 394·7)	65·2% (60·4 to 69·4)	12·9% (11·0 to 14·8)	21·3% (16·4 to 27·0)	4·5% (3·2 to 5·7)
	China	1059·7 (775·3 to 1433·2)	1233·9 (902·8 to 1668·8)	0·6% (0·5 to 0·8)	79·3% (65·6 to 89·5)	17·0% (7·7 to 30·9)	−1·3% (−4·4 to 1·9)
	Congo	9·5 (7·8 to 11·5)	474·8 (390·3 to 578·6)	26·9% (21·9 to 32·4)	45·3% (35·6 to 55·1)	26·6% (15·6 to 39·1)	4·9% (2·8 to 7·1)
	North Korea	70·9 (57·4 to 85·7)	1494·5 (1209·1 to 1806·1)	0%	95·5% (91·1 to 98·0)	4·4% (1·9 to 8·8)	1·9% (0·2 to 3·6)
	Democratic Republic of the Congo	69·5 (56·1 to 86·0)	177·3 (143·1 to 219·5)	30·5% (24·3 to 37·3)	12·7% (8·4 to 17·6)	52·5% (41·9 to 62·2)	3·0% (1·3 to 4·8)
	Ethiopia	126·5 (107·7 to 151·6)	550·7 (468·5 to 659·7)	23·6% (19·5 to 27·5)	41·5% (33·9 to 49·5)	24·0% (14·8 to 35·9)	2·5% (1·3 to 3·8)
	India	1871·8 (1421·4 to 2478·9)	644·0 (489·0 to 852·8)	5·9% (4·3 to 7·6)	47·7% (33·7 to 61·2)	43·8% (28·9 to 59·6)	7·9% (4·9 to 11·0)
	Indonesia	181·3 (145·3 to 226·3)	322·9 (258·7 to 403·1)	26·4% (20·9 to 32·5)	56·8% (45·8 to 66·9)	14·5% (6·4 to 26·8)	2·6% (0·3 to 4·8)
	Kenya	80·8 (69·5 to 95·6)	439·4 (378·2 to 520·1)	44·1% (37·0 to 50·9)	33·5% (25·9 to 41·3)	14·7% (7·5 to 25·1)	5·5% (2·8 to 8·0)
	Lesotho	23·5 (22·3 to 24·8)	900·7 (855·4 to 949·7)	70·1% (66·4 to 73·7)	12·2% (9·0 to 15·7)	17·7% (14·7 to 21·0)	12·8% (11·1 to 14·4)
	Liberia	2·9 (2·3 to 3·7)	258·2 (206·2 to 333·4)	33·1% (25·2 to 40·8)	33·1% (23·6 to 43·3)	30·9% (16·9 to 46·6)	5·0% (2·3 to 7·7)
	Mozambique	65·5 (55·9 to 77·0)	290·8 (248·1 to 341·5)	40·1% (33·9 to 46·6)	27·6% (21·8 to 33·6)	30·7% (20·1 to 41·0)	8·3% (7·0 to 9·5)
	Myanmar	52·9 (45·3 to 65·5)	486·2 (416·4 to 602·7)	58·5% (46·8 to 67·7)	15·8% (10·8 to 21·2)	25·7% (14·8 to 40·5)	9·5% (7·5 to 11·6)
	Namibia	88·6 (78·0 to 99·7)	4614·2 (4064·4 to 5191·2)	13·3% (11·8 to 15·0)	75·7% (71·5 to 79·5)	2·2% (1·1 to 3·8)	12·4% (10·2 to 14·3)
	Nigeria	263·7 (184·7 to 391·8)	448·0 (313·8 to 665·6)	27·7% (17·9 to 38·0)	19·3% (11·6 to 28·1)	51·6% (35·1 to 68·9)	5·6% (1·9 to 9·8)
	Pakistan	182·9 (139·5 to 248·3)	496·4 (378·8 to 674·0)	38·5% (27·7 to 49·4)	15·1% (9·6 to 21·4)	43·8% (28·5 to 58·7)	−0·6% (−2·8 to 1·8)
	Papua New Guinea	18·8 (16·3 to 21·9)	1249·1 (1085·9 to 1453·8)	40·2% (34·4 to 46·0)	57·5% (51·3 to 63·9)	2·3% (1·1 to 4·1)	15·4% (13·1 to 17·8)
	Philippines	214·4 (176·3 to 256·4)	903·7 (743·1 to 1080·8)	22·5% (18·7 to 27·1)	72·9% (67·0 to 77·8)	4·1% (1·8 to 7·8)	7·4% (5·4 to 9·4)
	Russia	2142·1 (1849·6 to 2472·4)	19 441·1 (16 786·1 to 22 438·0)	0·0%	90·0% (83·3 to 94·8)	9·2% (4·4 to 16·0)	4·9% (3·4 to 6·4)
	Sierra Leone	41·8 (37·6 to 46·2)	1770·4 (1591·4 to 1957·1)	21·5% (19·4 to 23·9)	71·6% (67·4 to 75·0)	3·4% (1·6 to 6·7)	9·1% (7·6 to 10·7)
	South Africa	405·2 (323·3 to 507·8)	935·7 (746·4 to 1172·6)	25·9% (20·4 to 32·0)	67·3% (59·3 to 74·4)	4·0% (1·7 to 7·5)	1·4% (−0·4 to 3·3)
	Thailand	39·5 (30·7 to 50·6)	676·9 (525·4 to 866·4)	23·3% (17·9 to 29·5)	64·5% (53·5 to 73·9)	10·7% (4·5 to 21·3)	1·3% (−1·1 to 3·8)
	Tanzania	102·7 (89·9 to 118·7)	481·9 (421·8 to 556·7)	32·3% (27·8 to 36·7)	20·8% (15·5 to 27·2)	38·9% (31·5 to 46·7)	6·1% (4·6 to 7·7)
	Vietnam	69·8 (55·6 to 89·0)	543·6 (433·4 to 693·3)	23·1% (17·9 to 28·6)	60·1% (48·1 to 69·8)	16·1% (7·4 to 29·1)	4·0% (2·0 to 6·0)
	Zambia	85·6 (81·3 to 90·8)	753·0 (715·3 to 799·4)	56·3% (53·0 to 59·3)	12·2% (9·0 to 16·1)	15·9% (12·9 to 19·0)	0·8% (0·3 to 1·3)
	Zimbabwe	40·2 (35·3 to 46·3)	301·4 (265·1 to 347·0)	34·1% (29·4 to 38·5)	19·6% (14·8 to 25·7)	37·0% (29·6 to 45·4)	3·9% (1·7 to 5·8)
Non-high tuberculosis burden low-income and middle-income countries							
	Afghanistan	28·8 (25·0 to 35·0)	702·7 (609·9 to 854·0)	58·5% (47·7 to 66·9)	19·6% (14·6 to 24·8)	21·9% (11·0 to 36·2)	9·6% (6·3 to 12·9)
	Albania	3·9 (2·8 to 5·5)	8983·0 (6476·1 to 12564·2)	0·9% (0·6 to 1·3)	98·7% (98·1 to 99·2)	0·3% (0·1 to 0·7)	8·7% (5·4 to 11·8)
	Algeria	32·5 (23·1 to 45·0)	2164·6 (1536·5 to 2999·1)	0%	99·8% (99·6 to 99·9)	0·2% (0·1 to 0·4)	7·2% (4·3 to 10·1)
	American Samoa	0·1 (0·1 to 0·1)	8940·0 (6081·1 to 12634·6)	0%	98·6% (97·3 to 99·4)	1·3% (0·5 to 2·6)	6·4% (3·2 to 9·6)
	Argentina	72·6 (50·3 to 105·8)	7206·6 (4991·7 to 10495·4)	0%	99·8% (99·7 to 99·9)	0·1% (0·0 to 0·2)	3·4% (0·4 to 6·4)
	Armenia	10·0 (8·2 to 11·9)	8917·4 (7297·9 to 10663·7)	20·1% (16·6 to 24·3)	76·2% (70·5 to 80·6)	3·7% (1·6 to 7·3)	9·6% (7·6 to 11·7)
	Azerbaijan	36·5 (28·4 to 47·5)	3187·1 (2478·4 to 4144·0)	20·5% (15·5 to 25·9)	46·8% (34·0 to 58·2)	32·5% (18·4 to 49·6)	10·9% (8·1 to 13·9)
	Belarus	107·5 (86·8 to 131·2)	29 128·9 (23 527·5 to 35 553·9)	2·9% (2·4 to 3·6)	97·0% (96·3 to 97·6)	0·1% (0·0 to 0·1)	2·8% (1·1 to 4·5)
	Belize	0·3 (0·3 to 0·4)	2265·5 (1933·3 to 2701·1)	52·6% (43·8 to 61·1)	43·7% (34·5 to 53·0)	3·4% (1·5 to 6·5)	7·4% (5·0 to 9·6)
	Benin	12·9 (9·9 to 16·9)	594·0 (459·3 to 778·7)	25·9% (19·5 to 33·0)	27·3% (19·3 to 36·1)	46·4% (33·3 to 60·0)	3·6% (1·2 to 6·2)
	Bhutan	2·4 (1·9 to 3·1)	2304·5 (1779·9 to 2911·1)	25·5% (19·8 to 32·4)	74·2% (67·2 to 79·9)	0·3% (0·1 to 0·6)	5·5% (2·9 to 8·1)
	Bolivia	20·1 (16·2 to 25·0)	2084·3 (1683·4 to 2601·0)	29·6% (23·4 to 36·2)	56·0% (46·0 to 66·1)	13·5% (6·4 to 23·3)	2·7% (0·7 to 4·5)
	Bosnia and Herzegovina	13·2 (9·3 to 17·8)	11040·1 (7794·3 to 14910·6)	0·1% (0·1 to 0·2)	99·1% (98·3 to 99·6)	0·8% (0·3 to 1·6)	6·3% (3·4 to 9·4)
	Botswana	72·4 (62·7 to 83·6)	4552·3 (3945·6 to 5259·7)	6·9% (6·0 to 7·9)	76·2% (72·3 to 79·8)	3·3% (2·3 to 4·5)	5·4% (4·3 to 6·6)
	Bulgaria	22·0 (15·9 to 30·2)	14 713·2 (10 631·2 to 20 131·2)	6·6% (4·7 to 8·9)	91·9% (88·6 to 94·4)	1·5% (0·6 to 3·0)	0·2% (−2·2 to 2·8)
	Burkina Faso	18·9 (14·4 to 25·1)	361·1 (275·5 to 479·4)	15·3% (11·3 to 19·6)	31·7% (22·2 to 42·1)	42·0% (27·6 to 56·1)	3·7% (1·7 to 5·6)
	Burundi	28·8 (27·0 to 30·8)	434·9 (407·0 to 465·4)	32·9% (30·7 to 35·1)	23·2% (20·7 to 26·0)	32·8% (28·8 to 36·9)	3·8% (3·1 to 4·5)
	Cabo Verde	1·2 (1·0 to 1·4)	1652·0 (1396·4 to 1951·6)	41·5% (34·9 to 48·8)	44·5% (35·7 to 54·0)	13·0% (6·5 to 22·5)	5·2% (3·2 to 7·3)
	Cameroon	39·6 (24·9 to 61·3)	559·3 (351·4 to 864·8)	9·3% (5·7 to 14·1)	13·9% (7·6 to 21·4)	72·3% (58·6 to 83·6)	3·6% (−0·3 to 7·5)
	Chad	23·4 (18·5 to 29·8)	544·0 (429·4 to 693·4)	8·7% (6·7 to 10·8)	32·1% (24·5 to 41·0)	57·2% (46·0 to 66·9)	2·6% (0·6 to 4·5)
	Colombia	76·4 (53·8 to 107·1)	6649·7 (4680·4 to 9328·4)	0·4% (0·3 to 0·5)	97·1% (94·7 to 98·6)	1·5% (0·6 to 3·3)	1·5% (−1·3 to 4·3)
	Comoros	1·0 (0·7 to 1·4)	594·1 (430·5 to 861·7)	37·0% (24·8 to 49·6)	6·5% (4·0 to 9·9)	54·5% (39·0 to 69·8)	3·9% (−0·1 to 7·7)
	Costa Rica	10·5 (7·1 to 14·6)	23 078·6 (15 649·6 to 32 005·4)	0%	99·8% (99·7 to 99·9)	0·2% (0·1 to 0·3)	10·1% (6·8 to 13·6)
	Cuba	28·2 (19·8 to 40·2)	36 718·7 (25 746·7 to 52 303·0)	0%	99·7% (99·5 to 99·9)	0·2% (0·1 to 0·5)	6·1% (3·0 to 9·3)
	Côte d'Ivoire	26·4 (20·9 to 34·6)	418·3 (332·1 to 548·3)	28·4% (21·3 to 35·2)	36·1% (25·8 to 46·8)	30·8% (16·5 to 46·7)	−0·6% (−3·3 to 2·0)
	Djibouti	3·4 (3·0 to 3·9)	834·3 (727·6 to 962·6)	54·2% (46·8 to 61·9)	33·7% (25·5 to 42·4)	11·8% (6·5 to 18·8)	6·6% (4·9 to 8·3)
	Dominica	0·1 (0·1 to 0·2)	5346·9 (4118·1 to 7159·2)	18·6% (13·7 to 23·7)	76·8% (69·4 to 82·9)	4·4% (1·9 to 8·8)	1·4% (−1·1 to 4·2)
	Dominican Republic	23·2 (17·0 to 31·7)	3735·1 (2742·4 to 5104·9)	5·7% (4·1 to 7·6)	91·8% (88·5 to 94·5)	1·9% (0·8 to 3·8)	6·5% (3·8 to 9·3)
	Ecuador	15·5 (11·0 to 22·0)	2773·6 (1964·1 to 3930·3)	0·1% (0·0 to 0·1)	95·3% (90·8 to 98·1)	4·1% (1·5 to 8·8)	2·1% (−0·8 to 4·8)
	Egypt	20·1 (14·6 to 27·0)	1079·8 (784·2 to 1450·8)	0·2% (0·2 to 0·3)	80·4% (65·3 to 90·1)	17·4% (8·0 to 31·7)	3·9% (1·2 to 6·6)
	El Salvador	7·9 (6·5 to 9·8)	4556·8 (3740·0 to 5626·1)	40·6% (32·5 to 48·9)	58·7% (50·3 to 66·8)	0·7% (0·3 to 1·4)	7·7% (5·4 to 10·1)
	Equatorial Guinea	11·2 (7·9 to 16·0)	2371·4 (1666·9 to 3406·3)	0·2% (0·1 to 0·2)	43·2% (28·2 to 59·4)	55·8% (39·4 to 71·1)	8·4% (4·5 to 12·0)
	Eritrea	13·1 (11·1 to 15·2)	450·5 (382·8 to 523·4)	9·5% (8·2 to 11·1)	10·4% (7·1 to 14·7)	76·9% (71·5 to 81·1)	1·7% (0·3 to 3·2)
	eSwatini	18·5 (17·0 to 20·4)	1692·0 (1556·5 to 1861·6)	41·7% (37·8 to 45·2)	22·7% (17·6 to 29·2)	27·4% (23·7 to 31·0)	14·5% (12·2 to 16·6)
	Federated States of Micronesia	0·0 (0·0 to 0·0)	320·6 (272·0 to 380·1)	53·4% (44·6 to 62·4)	45·9% (36·8 to 55·0)	0·8% (0·3 to 1·6)	5·4% (2·9 to 7·8)
	Fiji	3·9 (3·1 to 4·9)	11 592·0 (9155·1 to 14 586·8)	18·4% (14·5 to 23·0)	81·4% (76·8 to 85·4)	0·1% (0·0 to 0·2)	5·5% (3·3 to 7·7)
	Gabon	4·1 (3·0 to 5·4)	707·5 (522·6 to 928·5)	6·4% (4·7 to 8·4)	84·8% (77·1 to 90·0)	6·3% (2·7 to 12·9)	−1·9% (−4·2 to 0·5)
	Gambia	6·1 (5·9 to 6·5)	1057·0 (1012·2 to 1114·7)	73·7% (69·8 to 76·9)	16·5% (14·2 to 18·9)	7·9% (4·6 to 12·2)	9·0% (7·9 to 10·0)
	Georgia	22·9 (17·7 to 29·4)	8417·5 (6521·1 to 10785·9)	24·7% (19·0 to 31·4)	71·7% (64·4 to 78·6)	3·1% (1·4 to 6·2)	5·7% (3·2 to 8·3)
	Ghana	31·3 (25·0 to 40·9)	369·0 (295·2 to 481·7)	37·7% (28·4 to 46·4)	24·6% (16·8 to 32·6)	34·3% (21·0 to 50·0)	7·1% (3·5 to 10·7)
	Grenada	0·1 (0·1 to 0·2)	9505·0 (7266·6 to 12 327·9)	18·2% (13·8 to 23·4)	76·8% (69·5 to 82·6)	4·8% (2·0 to 9·4)	−3·2% (−5·5 to −0·6)
	Guatemala	8·1 (5·8 to 11·0)	2341·4 (1665·2 to 3157·0)	4·8% (3·5 to 6·6)	87·2% (79·7 to 92·3)	7·4% (3·1 to 14·8)	1·3% (−1·4 to 4·1)
	Guinea	9·0 (7·1 to 12·0)	277·0 (218·7 to 367·8)	36·8% (27·2 to 45·8)	17·5% (11·7 to 23·9)	40·5% (25·6 to 56·2)	3·8% (0·8 to 7·0)
	Guinea-Bissau	5·2 (4·6 to 6·2)	1080·8 (946·5 to 1280·7)	54·4% (45·6 to 61·7)	20·9% (16·8 to 24·9)	23·7% (14·4 to 35·6)	7·1% (4·9 to 9·4)
	Guyana	1·0 (0·8 to 1·3)	2186·0 (1683·6 to 2840·8)	24·8% (18·7 to 31·6)	74·8% (68·0 to 81·0)	0·4% (0·2 to 0·8)	5·6% (2·4 to 8·7)
	Haiti	1·7 (1·3 to 2·2)	182·7 (141·9 to 234·0)	11·9% (9·1 to 15·1)	86·6% (82·8 to 89·8)	1·5% (0·6 to 2·9)	4·6% (2·2 to 7·0)
	Honduras	6·2 (5·0 to 7·8)	1903·1 (1520·2 to 2374·3)	33·2% (26·3 to 41·1)	61·6% (52·1 to 69·8)	4·9% (2·0 to 9·6)	3·4% (1·3 to 5·6)
	Iran	75·9 (52·8 to 108·2)	5861·4 (4079·0 to 8350·8)	0%	99·4% (98·7 to 99·7)	0·6% (0·2 to 1·3)	9·2% (6·1 to 12·2)
	Iraq	32·2 (24·7 to 42·8)	1934·3 (1482·4 to 2574·7)	7·8% (5·8 to 10·0)	78·0% (67·0 to 86·0)	14·1% (6·6 to 25·7)	7·0% (4·1 to 9·7)
	Jamaica	4·8 (3·4 to 6·4)	10013·5 (7209·9 to 13421·2)	0%	86·9% (78·2 to 93·0)	9·3% (4·0 to 17·7)	−1·0% (−3·5 to 1·6)
	Jordan	3·6 (2·7 to 4·6)	3309·3 (2482·2 to 4317·4)	16·4% (12·3 to 21·5)	65·8% (53·7 to 75·6)	14·2% (6·3 to 26·4)	0·6% (−1·8 to 3·2)
	Kazakhstan	364·7 (293·2 to 451·0)	26 277·7 (21 124·6 to 32 499·7)	2·5% (2·0 to 3·1)	97·1% (96·3 to 97·7)	0·1% (0·0 to 0·1)	4·1% (2·6 to 5·7)
	Kiribati	1·0 (0·8 to 1·2)	3129·0 (2580·7 to 3750·4)	30·6% (25·3 to 36·8)	69·2% (63·0 to 74·6)	0·2% (0·1 to 0·3)	4·1% (2·3 to 6·0)
	Kyrgyzstan	51·5 (46·2 to 57·2)	7255·9 (6512·9 to 8062·4)	22·3% (20·0 to 24·7)	67·4% (62·2 to 71·5)	10·3% (6·4 to 15·9)	8·1% (7·0 to 9·3)
	Laos	6·7 (5·5 to 8·2)	677·3 (553·8 to 826·9)	25·8% (20·9 to 31·3)	59·5% (50·4 to 67·9)	13·7% (6·8 to 23·9)	2·7% (0·6 to 4·7)
	Lebanon	7·8 (5·7 to 10·4)	4767·7 (3502·4 to 6398·0)	7·6% (5·5 to 10·0)	79·9% (70·3 to 86·9)	8·9% (3·9 to 17·4)	−0·1% (−2·8 to 2·6)
	Libya	8·4 (5·9 to 11·7)	4357·4 (3044·3 to 6073·7)	0%	99·2% (98·4 to 99·6)	0·6% (0·2 to 1·3)	0·4% (−2·5 to 3·4)
	Madagascar	7·0 (5·6 to 8·9)	142·4 (112·8 to 181·1)	17·4% (13·5 to 21·6)	44·3% (32·7 to 56·4)	25·7% (14·2 to 40·0)	3·3% (1·0 to 5·7)
	Malawi	28·6 (26·7 to 30·7)	302·6 (283·1 to 325·1)	65·2% (60·6 to 69·6)	9·1% (6·4 to 12·5)	24·2% (19·8 to 28·8)	6·8% (6·2 to 7·5)
	Malaysia	88·5 (64·0 to 118·5)	4790·6 (3463·6 to 6414·0)	0%	98·9% (97·8 to 99·5)	0·9% (0·4 to 1·9)	7·8% (5·1 to 10·7)
	Maldives	1·3 (0·9 to 1·8)	6871·2 (4726·5 to 9655·6)	0%	98·2% (96·6 to 99·3)	1·3% (0·5 to 2·8)	5·5% (2·6 to 8·4)
	Mali	13·9 (10·4 to 19·1)	495·1 (368·6 to 681·1)	0·4% (0·3 to 0·5)	39·3% (25·9 to 52·8)	56·4% (42·0 to 70·4)	4·9% (1·5 to 8·5)
	Marshall Islands	0·8 (0·6 to 1·1)	8274·1 (5946·7 to 11044·7)	4·3% (3·1 to 5·8)	95·1% (93·3 to 96·5)	0·5% (0·2 to 1·0)	5·2% (2·5 to 7·9)
	Mauritania	2·2 (1·7 to 3·0)	501·5 (386·2 to 679·9)	28·6% (20·7 to 36·5)	31·7% (21·3 to 43·2)	37·7% (23·4 to 54·8)	8·5% (4·5 to 12·2)
	Mauritius	1·3 (0·9 to 1·7)	5012·6 (3649·6 to 6767·4)	8·2% (5·9 to 10·9)	86·9% (80·8 to 91·5)	4·9% (1·9 to 9·8)	5·1% (2·5 to 7·8)
	Mexico	57·6 (38·1 to 79·4)	2643·5 (1747·7 to 3645·4)	0·8% (0·6 to 1·2)	99·0% (98·5 to 99·3)	0·1% (0·0 to 0·1)	2·5% (−0·4 to 5·4)
	Mongolia	16·5 (13·9 to 19·6)	3240·0 (2729·2 to 3854·2)	20·1% (16·7 to 23·6)	72·5% (66·8 to 77·7)	7·1% (3·7 to 12·0)	8·2% (6·3 to 10·3)
	Montenegro	1·1 (0·8 to 1·5)	11 247·2 (7758·0 to 15 591·0)	0·4% (0·3 to 0·5)	98·9% (98·1 to 99·4)	0·7% (0·3 to 1·4)	−0·3% (−2·9 to 2·4)
	Morocco	32·6 (24·1 to 43·5)	639·6 (471·9 to 851·5)	3·8% (2·8 to 5·0)	78·0% (63·8 to 87·4)	17·6% (8·3 to 31·9)	3·5% (0·5 to 6·4)
	Nepal	20·4 (15·9 to 26·3)	421·3 (328·0 to 541·8)	2·1% (1·6 to 2·7)	70·5% (56·5 to 81·5)	25·3% (14·0 to 39·9)	3·9% (1·3 to 6·4)
	Nicaragua	8·4 (6·8 to 10·4)	3967·3 (3218·1 to 4941·3)	36·4% (28·9 to 44·3)	63·1% (55·2 to 70·7)	0·5% (0·2 to 1·0)	13·8% (11·3 to 16·5)
	Niger	5·2 (3·1 to 8·3)	131·6 (79·6 to 211·2)	7·9% (4·6 to 12·2)	20·0% (10·7 to 32·4)	70·7% (54·8 to 83·9)	3·5% (−0·9 to 8·2)
	North Macedonia	2·0 (1·5 to 2·7)	3555·7 (2593·7 to 4803·2)	4·1% (2·9 to 5·4)	83·2% (72·9 to 89·9)	11·6% (5·2 to 21·7)	−0·8% (−3·3 to 1·8)
	Palestine	2·2 (1·6 to 3·1)	3536·2 (2494·8 to 4922·7)	0%	92·8% (86·1 to 96·9)	4·5% (1·7 to 9·3)	5·6% (2·6 to 8·5)
	Paraguay	11·7 (8·9 to 15·0)	3451·6 (2640·1 to 4443·3)	16·6% (12·7 to 21·3)	71·4% (61·0 to 79·6)	10·8% (4·8 to 20·6)	6·7% (4·4 to 9·3)
	Peru	131·6 (96·1 to 174·9)	4656·0 (3400·0 to 6184·8)	1·9% (1·4 to 2·5)	95·5% (92·7 to 97·2)	2·4% (1·0 to 4·9)	4·6% (2·3 to 7·0)
	Moldova	41·7 (36·0 to 47·9)	12 800·8 (11 051·7 to 14 718·6)	17·6% (15·2 to 20·2)	79·0% (75·4 to 82·1)	2·4% (1·1 to 4·5)	10·4% (8·7 to 12·4)
	Romania	104·8 (76·3 to 145·7)	8381·1 (6099·1 to 11 645·9)	3·9% (2·8 to 5·3)	95·9% (94·5 to 97·1)	0·2% (0·1 to 0·4)	0·9% (−1·7 to 3·7)
	Rwanda	21·7 (19·6 to 24·0)	471·6 (425·3 to 522·8)	41·7% (37·6 to 46·2)	34·6% (28·8 to 40·8)	13·0% (9·6 to 17·3)	4·7% (3·9 to 5·6)
	Saint Lucia	0·2 (0·1 to 0·3)	7021·3 (5222·3 to 9528·6)	11·5% (8·3 to 15·1)	83·3% (76·7 to 88·3)	4·8% (1·9 to 10·0)	1·9% (−0·7 to 4·8)
	Saint Vincent and the Grenadines	0·1 (0·1 to 0·2)	4965·3 (3727·4 to 6448·5)	17·9% (13·5 to 23·4)	77·5% (70·4 to 83·2)	4·3% (1·9 to 8·8)	2·0% (−0·4 to 4·6)
	Samoa	0·1 (0·1 to 0·2)	1784·3 (1327·3 to 2351·3)	14·2% (10·6 to 18·7)	78·2% (70·2 to 84·6)	5·0% (2·0 to 10·6)	2·0% (−0·6 to 4·6)
	São Tomé and Príncipe	0·8 (0·8 to 0·9)	4124·1 (3806·9 to 4522·6)	46·5% (42·3 to 50·2)	19·1% (14·0 to 25·2)	7·0% (3·1 to 13·3)	−6·7% (−8·7 to −4·1)
	Senegal	11·1 (8·9 to 14·0)	366·6 (294·6 to 461·8)	30·6% (24·0 to 37·6)	41·7% (31·5 to 52·5)	24·4% (12·1 to 40·0)	3·4% (0·7 to 6·0)
	Serbia	17·4 (12·3 to 24·2)	7876·0 (5569·0 to 10 975·2)	0·1% (0·0 to 0·1)	96·6% (93·2 to 98·6)	3·3% (1·3 to 6·6)	3·5% (0·8 to 6·1)
	Solomon Islands	2·2 (2·0 to 2·4)	4707·5 (4223·9 to 5255·0)	59·9% (53·5 to 66·5)	39·8% (33·1 to 46·3)	0·3% (0·1 to 0·5)	8·5% (6·6 to 10·6)
	Somalia	22·0 (20·9 to 23·2)	433·9 (412·6 to 458·0)	63·6% (60·2 to 66·9)	7·8% (6·5 to 9·1)	27·9% (24·4 to 31·6)	8·5% (7·5 to 9·6)
	South Sudan	19·8 (18·5 to 21·1)	630·4 (588·7 to 673·0)	40·2% (37·6 to 43·0)	6·2% (5·0 to 7·8)	47·0% (43·3 to 50·6)	7·2% (5·6 to 9·1)
	Sri Lanka	18·6 (13·1 to 25·8)	1855·0 (1303·6 to 2568·8)	9·2% (6·4 to 12·6)	88·5% (84·0 to 92·1)	2·1% (0·9 to 4·4)	−7·6% (−10·3 to −5·1)
	Sudan	9·9 (7·7 to 13·1)	274·9 (213·4 to 362·7)	41·7% (31·1 to 52·8)	24·2% (15·9 to 33·4)	32·6% (16·8 to 50·2)	5·7% (2·8 to 8·8)
	Suriname	1·0 (0·9 to 1·1)	8293·2 (7492·4 to 9371·3)	69·5% (61·3 to 76·6)	30·1% (22·9 to 38·4)	0·2% (0·1 to 0·4)	8·1% (5·8 to 10·4)
	Syria	2·5 (2·0 to 3·2)	655·4 (511·1 to 824·5)	23·2% (18·1 to 29·3)	59·8% (49·4 to 69·4)	16·1% (8·1 to 27·2)	−0·2% (−2·6 to 2·3)
	Tajikistan	28·9 (25·7 to 33·1)	2936·5 (2616·0 to 3363·8)	55·7% (48·5 to 62·3)	30·4% (24·1 to 37·5)	13·8% (7·6 to 22·8)	10·6% (9·0 to 12·1)
	Timor-Leste	3·7 (3·0 to 4·5)	1782·6 (1470·9 to 2182·0)	46·4% (37·6 to 55·7)	53·0% (43·6 to 62·0)	0·4% (0·2 to 0·9)	8·6% (6·6 to 10·7)
	Togo	5·4 (4·1 to 7·3)	295·7 (227·8 to 400·2)	36·0% (26·0 to 45·7)	18·6% (12·0 to 26·0)	40·1% (25·4 to 57·2)	4·9% (1·7 to 8·3)
	Tonga	0·2 (0·1 to 0·2)	5516·1 (4200·9 to 7193·2)	9·5% (7·1 to 12·2)	68·3% (56·5 to 78·7)	21·5% (11·7 to 34·0)	4·1% (1·5 to 6·9)
	Tunisia	1·6 (1·2 to 2·3)	517·1 (360·8 to 719·1)	0·3% (0·2 to 0·5)	94·5% (89·3 to 97·4)	5·0% (2·0 to 10·0)	4·7% (1·6 to 7·7)
	Turkey	65·5 (45·8 to 93·5)	2868·0 (2003·2 to 4092·6)	0·0% (0·0 to 0·0)	97·3% (94·5 to 98·8)	2·4% (1·0 to 5·2)	0·9% (−1·7 to 3·9)
	Turkmenistan	17·2 (13·4 to 21·7)	4215·3 (3288·0 to 5315·7)	14·0% (10·9 to 17·7)	66·9% (53·0 to 76·7)	18·5% (9·2 to 32·3)	6·9% (4·7 to 9·1)
	Uganda	53·1 (43·4 to 68·3)	261·1 (213·5 to 335·5)	39·4% (30·2 to 47·5)	17·4% (12·4 to 23·4)	40·8% (28·2 to 54·1)	2·9% (1·2 to 4·8)
	Ukraine	250·9 (205·1 to 310·0)	8070·6 (6596·6 to 9972·3)	6·5% (5·2 to 7·9)	88·6% (84·3 to 91·8)	4·8% (2·2 to 9·2)	4·1% (2·1 to 6·3)
	Uzbekistan	115·9 (93·4 to 141·5)	5929·1 (4774·1 to 7236·6)	11·7% (9·5 to 14·4)	67·9% (57·2 to 76·3)	20·2% (12·0 to 31·0)	9·6% (7·5 to 11·7)
	Vanuatu	0·5 (0·4 to 0·7)	2919·0 (2168·6 to 3906·1)	3·3% (2·4 to 4·3)	92·4% (88·7 to 95·0)	3·8% (1·7 to 6·8)	4·8% (2·2 to 7·6)
	Venezuela	7·6 (5·4 to 10·3)	1036·5 (736·1 to 1404·5)	0·0% (0·0 to 0·0)	89·5% (80·4 to 95·0)	5·6% (2·3 to 11·5)	−5·6% (−8·2 to −2·8)
	Yemen	3·0 (2·0 to 4·5)	181·3 (123·0 to 274·7)	23·2% (14·7 to 32·8)	23·3% (13·6 to 35·1)	52·9% (34·6 to 70·8)	−3·1% (−6·1 to 0·1)

Data in parentheses are 95% CIs. Spending is presented in inflation-adjusted 2019 US$. Notified treated cases were reported by the ministry of health from each country to WHO. Countries are listed alphabetically in each tuberculosis burden group. Tuberculosis DAH for 135 low-income and middle-income countries includes spending on two parts: administration and global projects and country projects. However, tuberculosis DAH only includes spending on country-specific projects for country groups and individual countries. DAH=development assistance for health. GBD=Global Burden of Diseases, Injuries, and Risk Factors study.

* India and China are presented separately because they are the top two high tuberculosis burden countries.

Increases in spending were driven by government spending on notified cases of tuberculosis (figure 1), which increased from $2·3 billion (95% CI 2·2–2·3) in 2000 to $5·8 billion (5·6–6·1) in 2017. Spending by governments on non-notified cases increased from $0·8 billion (0·6–1·0) in 2000 to $1·1 billion (0·8–1·6) in 2017, such that overall government spending for notified and non-notified cases combined increased by 5·1% (4·4–5·7) per year, reaching $6·9 billion (6·5–7·5) in 2017. The annualised growth rate of government spending on tuberculosis was 11·3% (95% CI 8·7–14·0) for India and 4·7% (1·8–7·8) for China, two countries with the highest burden of tuberculosis. Total out-of-pocket spending decreased from $2·4 billion (1·9–3·1) in 2000 to $2·1 billion (1·6–2·7) in 2017. For India, out-of-pocket spending was $357·7 million (188·6–606·6) in 2000, $775·6 million (424·1–1289·6) in 2014, and $836·2 million (435·0–1411·8) in 2017. In China, out-of-pocket spending was $831·4 million (421·6–1487·0) in 2000, $204·7 million (90·0–412·0) in 2014, and $180·1 million (78·7–349·2) in 2017. The total amount of tuberculosis spending from prepaid private sources (eg, private health insurance) remained relatively small throughout 2000–17, at $246·9 million (171·9–368·7) in 2000 and $225·0 million (184·1–280·7) in 2017. Development assistance for tuberculosis for country-specific projects substantially increased from $54·6 million in 2000 to $1·1 billion in 2017. The Global Fund to Fight AIDS, Tuberculosis and Malaria spent $893·6 million on country-specific tuberculosis projects in 2017, an increase of $891·2 million from 2002 (first year with DAH data), and the US government spent $98·3 million on country-specific tuberculosis projects in 2017, an increase of $91·6 million from 2000. DAH spending on administrative costs and global initiatives for tuberculosis prevention and care that cannot be disaggregated by country increased from $85·3 million in 2000 to $576·2 million in 2017.

Although absolute prepaid private and out-of-pocket spending decreased over time, the increases in development assistance for tuberculosis and government spending changed the relative contribution of each source. From 2000 to 2017, the proportion of spending accounted for by total DAH increased from 2·4% (95% CI 2·2–2·7) to 15·8% (14·7–16·8). Meanwhile, the proportion accounted for by government health spending for notified and non-notified cases combined increased from 52·3% (46·0–57·4) in 2000 to 63·5% (59·2–66·8) in 2017. Total government health spending on tuberculosis increased by 131·6% (95% CI 108·8–157·3) from $3·0 billion (2·8–3·2) in 2000 to $6·9 billion (6·5–7·5) in 2017. Comparatively, the proportion of out-of-pocket spending decreased from 40·9% (35·4–48·2) to 18·7% (15·2–23·6), and the proportion of prepaid private spending decreased from 4·3% (2·9–6·4) to 2·1% (1·7–2·6). We present results from sensitivity analyses to illustrate the influence of assumptions on our results in terms of mean estimates and confidence intervals (pp 28–30). When we tested a scenario in which 75% of non-notified cases were treated, our estimate of total tuberculosis spending for 2017 was not significantly different from the primary result for which we assumed all non-notified cases were treated.

The increase in spending was driven by national tuberculosis programme spending, which increased from $1·2 billion in 2000, to $4·8 billion in 2017 (figure 1B). Outpatient spending increased from $1·3 billion (1·2–1·6) in 2000, to $2·5 billion (2·1–3·0) in 2017. Inpatient spending increased from $1·5 billion (1·3–1·9) in 2000, to $2·1 billion (1·7–2·5) in 2017. Private drug spending decreased from $1·5 billion (1·3–1·9) in 2000 to $1·1 billion (0·9–1·3) in 2017. In 2017, 43·5% (40·4–46·3) of total spending was on the national tuberculosis programme, 22·7% (20·4–25·6) was on outpatient services, 18·8% (16·2–21·8) was on inpatient services, 9·7% (8·4–11·3) was on non-national tuberculosis programme drugs, and 5·3% (4·9–5·6) was on development assistance for tuberculosis on administrative costs and global initiatives for tuberculosis prevention and care.

**Figure 2:**
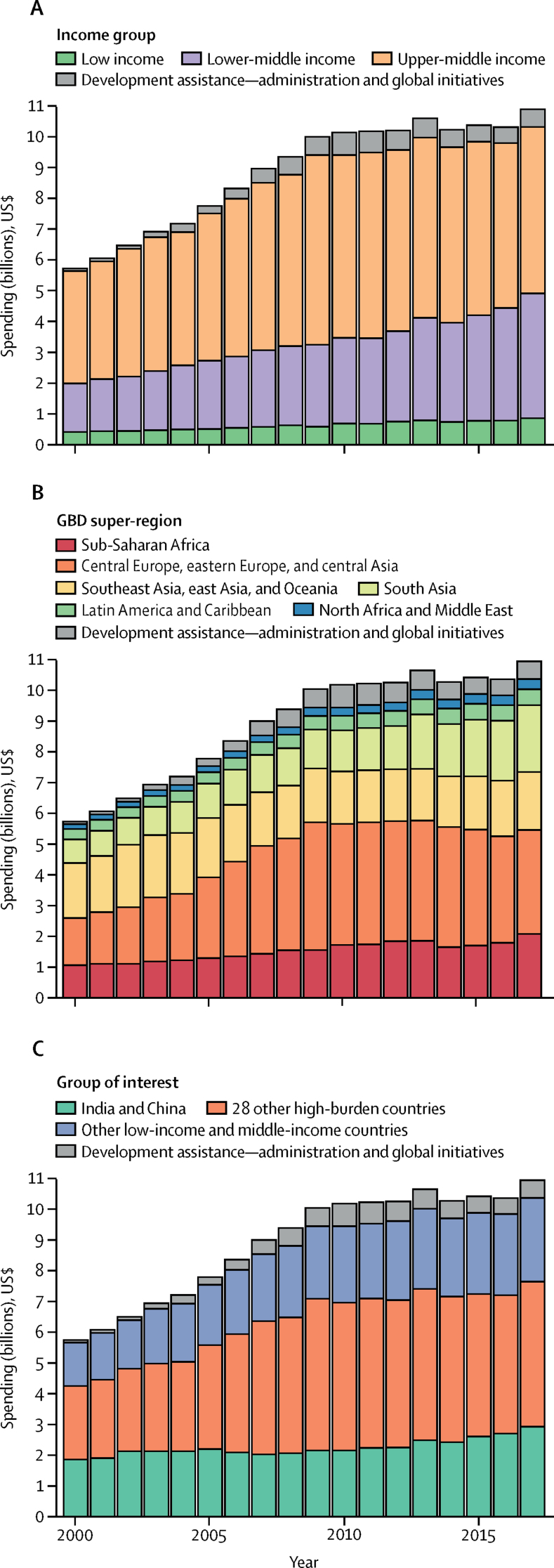
Tuberculosis spending in low-income and middle-income countries by 2019 World Bank income group (A), GBD super-region (B), and WHO defined high tuberculosis burden country groups (C), 2000–17 Spending estimates are presented in inflation-adjusted 2019 US$. GBD=Global Burden of Diseases, Injuries, and Risk Factors study.

Tuberculosis spending in low-income and middle-income countries in 2017 by income group, region, and tuberculosis burden categories (as defined by WHO for the period 2016–20) is shown in figure 2. In 2017, among income groups, upper-middle-income countries accounted for the largest share of total spending on tuberculosis at $5·4 billion (95% CI 5·0–6·0) followed by lower-middle-income countries at $4·1 billion (3·6–4·7; table 2). Low-income countries accounted for a relatively small amount of tuberculosis spending, from $423·8 million (403·2–446·8) in 2000 to $872·0 million (833·9–913·3) in 2017. Among six GBD super-regions, tuberculosis spending was highest in southeast Asia, east Asia, and Oceania in 2000, at $1·8 billion (1·3–2·5). However, in 2017, central Europe, eastern Europe, and central Asia (with Russia having the highest spending on tuberculosis of all low-income and middle-income countries) spent $3·4 billion (3·1–3·7) compared with $1·9 billion (1·6–2·3) spent in southeast Asia, east Asia, and Oceania (table 2). Among countries defined by WHO as high tuberculosis burden countries for the period 2016–20, the 30 high tuberculosis burden countries accounted for 81·9% (81·4–82·2) of global tuberculosis incidence and 73·7% (71·8–75·8) of tuberculosis spending in 2017 (figure 2C). India and China are of particular interest because among the top 30 countries, at least 40% of incident cases are in these two countries,^1^, ^2^ accounting for 28·2% (24·1–33·2) of tuberculosis spending in 2017 (figure 2).

**Figure 3:**
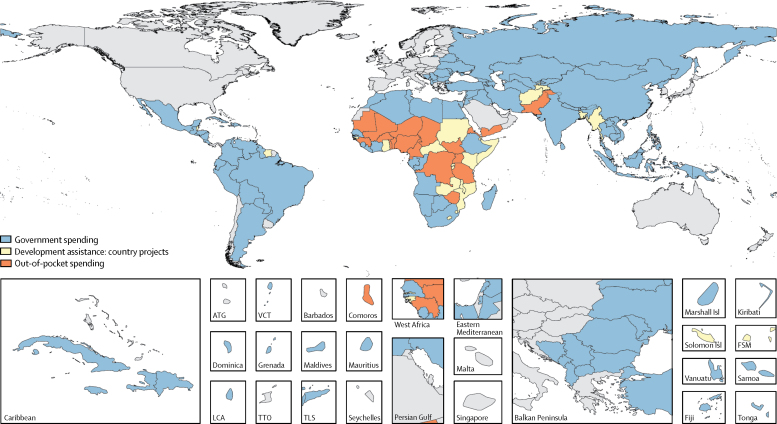
Primary financing source for total spending on tuberculosis in low-income and middle-income countries, 2017 Areas that are unshaded do not have estimates or are not low-income or middle-income countries. ATG=Antigua and Barbuda. FSM=Federated States of Micronesia. Isl=islands. LCA=Saint Lucia. TLS=Timor-Leste. TTO=Trinidad and Tobago. VCT=Saint Vincent and the Grenadines.

The primary source of funding across all 135 low-income and middle-income countries in 2017 is shown in figure 3, with further spending breakdowns shown in table 2. DAH was the primary financing source for 24 (18%) low-income and middle-income countries, government spending was the primary source in 91 (67%) countries, and out-of-pocket spending was the primary source in 20 countries (15%). Among the 30 high tuberculosis burden countries, 18 (60%) countries (all six upper-middle-income countries, eight lower-middle-income countries, and four low-income countries) had government spending as the primary financing source (high tuberculosis burden countries by World Bank income group and primary spending are shown in the appendix [p 10]). In North Korea, Brazil, and Russia, government spending accounted for 90% or more of total tuberculosis spending (table 2). Government spending on tuberculosis was 47·7% (95% CI 33·7–61·2) in India and 79·3% (65·6–89·5) in China in 2017. Seven (23%) high tuberculosis burden countries (including all five lower-middle-income countries, and two low-income countries) had development assistance for tuberculosis as their primary financing source (appendix p 10). The proportion of total spending accounted for by DAH in Lesotho was 70·1% (95% CI 66·4–73·7), in Central African Republic was 65·2% (60·4–69·4), and in Myanmar was 58·5% (46·8–67·7). Five (17%) high tuberculosis burden countries (including all three lower-middle-income countries, and two low-income countries) had out-of-pocket spending as their primary source of tuberculosis spending (appendix p 10). The proportion of tuberculosis spending accounted for by out-of-pocket spending in Democratic Republic of the Congo was 52·5% (41·9–62·2), in Nigeria was 51·6% (35·1–68·9), and in Pakistan was 43·8% (28·5–58·7).

**Figure 4:**
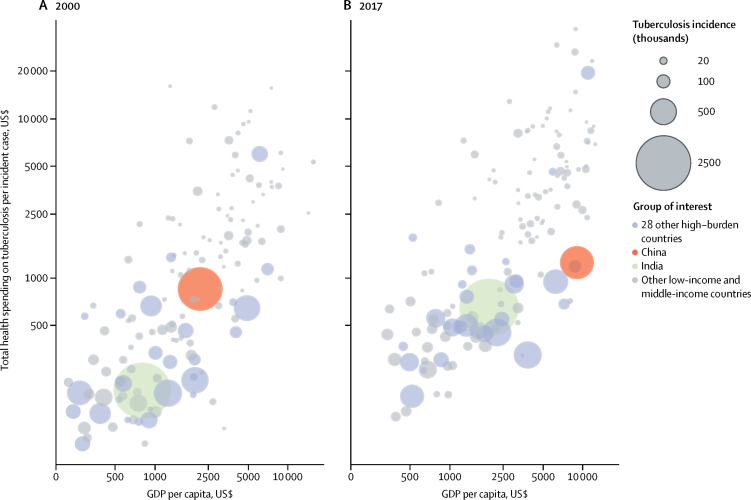
Total spending on tuberculosis per incident case, by GDP per capita, for 2000 (A) and 2017 (B) Total spending on tuberculosis per incident case estimates are presented in inflation-adjusted 2019 US$. GDP=gross domestic product.

Total spending on tuberculosis per incident case by GDP per capita for 2000 and 2017 is shown in figure 4. Overall, we found a positive correlation between spending per incident case and GDP. In India, the country with the highest estimated incidence of tuberculosis globally (about a quarter of all global cases—ie, 2·907 million [2·651–3·191] cases in 2017), spending per incident case substantially increased from $193 (95% CI 128–286) in 2000 to $644 (489–853) in 2017, with GDP per capita increasing from $793 (742–835) in 2000 to $1954 (1925–1978) in 2017; while in China—the country with the second highest estimated number of incident cases per year globally (ie, 858·8 thousand [783·4–942·0] cases in 2017)—spending per incident case slightly increased from $843 (553–1279) to $1234 (903–1669), whereas GDP per capita increased from $2174 (2152–2196) in 2000 to $8990 (8882–9086) in 2017. Among the other 28 high tuberculosis burden countries, Russia had the highest total spending per incident case, at $5995 (4865–7360) in 2000 and $19 441 (16 786–22 438) in 2017, with GDP per capita increasing from $6107 (6057–6144) in 2000 to $10 902 (10 729–11 050) in 2017 (table 2). Average spending per incident case was $655 (580–755) in lower-middle-income countries and $411 (393–430) in low-income countries; whereas average sending per incident case in upper-middle-income countries was $2944 (2697–3226).

## Discussion

We estimated that in 2017, $10·9 billion (95% CI 10·3–11·8) was spent on tuberculosis in low-income and middle-income countries. Government spending amounted to $6·9 billion (6·5–7·5), or 63·5% (59·2–66·8), of all tuberculosis spending in 2017. Overall, this study indicates a decreasing reliance on out-of-pocket spending, with increased reliance on government spending and development assistance for tuberculosis. The estimates of tuberculosis spending by source and function can be used by different stakeholders.

By contrast with spending for other infectious diseases, most country-specific tuberculosis spending was in upper-middle-income countries, in spite of their relatively low burden of tuberculosis cases (18·1% of global incidence).^14^ Spending was much higher in the upper-middle-income group because of higher costs ($2944 per incident case), compared with $655 in lower-middle-income countries and $411 in low-income countries).

From 2000 to 2017, tuberculosis spending sourced from governments increased by 131·6%, from $3·0 billion to $6·9 billion, suggesting that countries are relying more on government resources to cover tuberculosis care than they have done previously. For instance, government spending on tuberculosis increased at an annualised rate of 11·3% in India and 4·7% in China. Increases in government spending on tuberculosis between 2000 and 2017 are consistent with the global tuberculosis strategies and targets recommended by WHO and adopted by countries during this period. From 2000 to 2005, national tuberculosis programmes focused on implementation of the DOTS^15^ and achievement of targets to detect 70% of sputum smear-positive pulmonary cases (ie, those with the most infectious form of the disease) and to successfully treat 85% of these cases. From 2006 to 2015, efforts in tuberculosis prevention, diagnosis, and treatment broadened considerably, based on the Stop TB Strategy.^16^ This strategy set targets to reduce the incidence rate and to halve tuberculosis prevalence and mortality rates between 1990 and 2015. Since 2016, national and global efforts have been undertaken within the framework of the End TB Strategy.^3^ Between 2000 and 2017, the annual number of notified cases of tuberculosis in low-income and middle-income countries increased from 3·6 million in 2000 to 6·2 million in 2017, and the number of people treated for multidrug-resistant tuberculosis increased from negligible levels to 140 000 in 2017.^2^

Our estimates of government spending are similar to those previously published by WHO.^2^ Estimated domestic funding for notified cases in 2017 was $5·2 billion in the latest Global Tuberculosis Report,^2^ and $5·8 billion in this study. Two main reasons exist for the difference: we included 135 low-income and middle-income countries in this study, whereas the Global Tuberculosis Report included estimates for 119 of these countries; and we included pre-diagnosis spending, which the Global Tuberculosis Report did not.

To our knowledge, this is the first study to model out-of-pocket spending on tuberculosis across all low-income and middle-income countries and across time. In our out-of-pocket spending estimation, we included all direct medical costs borne by the patient with tuberculosis, including pre-diagnosis, health-care, and private drug costs. We estimated that $2·1 billion was spent out of pocket in 2017, including on inpatient and outpatient services and private drugs. Our modelled estimates of out-of-pocket spending are consistent with other literature that shows that a large proportion of tuberculosis spending is paid out of pocket in many countries. For example, Arinaminpathy and colleagues^17^ used data on drug sales to estimate that over US$59 million was spent out of pocket on first-line tuberculosis drugs alone in India in 2014, and similar findings were presented in trend analyses.^18^ In comparison with Arinaminpathy and colleagues' study, our estimates are that in India in 2014 out-of-pocket spending was $775·6 million, including health-care use and first-line and second-line drugs. Even among countries with strong public-sector services for tuberculosis, out-of-pocket spending has been observed. We estimated that out-of-pocket spending accounted for 9·2% of spending for tuberculosis in Russia in 2017, a country that is generally assumed to have only public-sector engagement in tuberculosis care. Overall reductions in out-of-pocket spending highlight improvements in notification of tuberculosis cases and the ability of public systems to finance tuberculosis care and treatment.

We estimated DAH for country-specific tuberculosis projects to be $1·1 billion in 2017, a large increase since 2000. Most of this increase in spending came from the Global Fund to Fight AIDS, Tuberculosis and Malaria and the US Government. Because of the impermanence of DAH for disease control programmes, countries will need to diversify their sources of funding to meet spending targets.

We would like to highlight trade-offs that accompany the move from reliance on out-of-pocket spending to reliance on national tuberculosis programme spending, since the composition of total incident cases has shifted to an increase in the proportion of notified cases over time. The decrease in out-of-pocket spending is explained by the fact that more incident cases have been notified over the period 2000 to 2017, which drives the modelled out-of-pocket estimates.

Our study had several limitations. First, little data were available about the proportion of unreported patients who had been treated. Based on a 2016 study from India,^17^ which accounts for a large share of the global number of non-notified cases, we assumed that all unreported cases were treated on the basis of the assumption (discussed with global experts on tuberculosis) that most people with tuberculosis will seek care or self-medicate in the public or private sector. Results from our sensitivity analysis in which we used an alternative assumption (ie, 75% of non-notified cases were treated) support the results in the main analysis. Second, although data on use of general health services were available for notified cases from the WHO Global Tuberculosis database, we did not have data about outpatient and inpatient use of health-care services before or during treatment for non-notified cases. We assumed that service use for non-notified cases was the same as for notified cases, which could lead to an underestimation or overestimation of health-care spending among non-notified cases. Third, given the absence of data on non-national tuberculosis programme drug spending, we used the tuberculosis drug cost per patient from the national tuberculosis programme as the drug cost for non-notified patients. But given the ability of the government to buy drugs in bulk, and the increased mark-up of price and health-care price in the unengaged private sector, our out-of-pocket drug costs have likely been underestimated. Fourth, we did not include spending on interventions related to tuberculosis prevention, such as immunisation and preventive treatment for a latent tuberculosis infection (especially among patients with HIV). Funding for tuberculosis research and development from major pharmaceutical funders and philanthropists who fund most research, rather than development work, was also excluded given the focus of our study on modelling finances for prevention, diagnosis, and treatment for tuberculosis. Estimating spending on these interventions is an interesting topic to cover in future research. Finally, directly observed treatment might not require the same level of resources as an average outpatient visit but adjusting the available unit cost estimates to account for these treatments specifically is a challenge. We might have overestimated the cost of a directly observed treatment visit.

Estimating tuberculosis spending by financing source and function in all low-income and middle-income countries provides comparable information across countries and over time. To our knowledge, this study provides the first estimates of total spending on care and treatment of tuberculosis that include out-of-pocket and prepaid private spending. This research also presents the first estimates of spending on non-notified tuberculosis cases. Our aggregated estimates by income group, region, and tuberculosis-burden category provide crucial insights for countries on the path to elimination of tuberculosis. This study provides quantified evidence on spending in 2017. Despite substantial increases in national tuberculosis programme spending, our estimates of government tuberculosis spending in 2017, at $6·9 billion, was $2·3 billion short of the 2017 spending target of $9·2 billion in the Global Plan set by the Stop TB Partnership and was less than half of the global target agreed on by all UN member states that has been set for 2022. The estimates in this study can be used to assess past programmes and serve as a baseline for future investments in tuberculosis.

## Data sharing

Data used for this study were extracted from publicly available sources that are listed in the appendix (p 8). All estimates from this study are available on the Global Health Data Exchange website.
