# Supplementary material for: Tracking total spending on tuberculosis by source and function in 135 low-income and middle-income countries, 2000–17: a financial modelling study
Source: Lancet Infect Dis. 2020 Aug;20(8):929–42. doi: 10.1016/S1473-3099(20)30124-9 (PMC7649746; doi:10.1016/S1473-3099(20)30124-9)
Supplement: Supplementary appendix [file mmc1.pdf]

THE LANCET
Infectious Diseases

Supplementary appendix

This appendix formed part of the original submission and has been peer reviewed. We post it as supplied by the authors.

Supplement to: Su Y, Baena IG, Harle AC, et al. Tracking total spending on tuberculosis by source and function in 135 low-income and middle-income countries, 2000–17: a financial modelling study. *Lancet Infect Dis* 2020; published online April 23. http://dx.doi.org/10.1016/S1473-3099(20)30124-9.

Supplementary Methods Appendix

Tracking total spending on tuberculosis by source and function in 135 low- and middle-income countries, 2000–2017: a financial modeling study

Version: Jan 31st, 2020

Contents

S1. Summary statistics 2

S2. Estimating tuberculosis domestic spending 11

S2.1. Modeling methods: ST-GPR 11

S2.2. Data sources, assumptions, and estimates 12

S2.2.1. Pre-treatment 13

S2.2.2. **Notified cases** 14

S2.2.3. Non-notified cases 18

S2.3. Modeling financing sources and functions 22

S2.3.1. Modeling total government spending on tuberculosis 22

S2.3.2. Modeling total out-of-pocket spending on tuberculosis 23

S2.3.3. Modeling prepaid private spending on tuberculosis 23

S2.3.4. Modeling spending on tuberculosis by function 24

S3. Currency Conversion 27

S4. Sensitivity analysis 28

S5. Results in purchasing power parity (PPP) dollars 31

S6. How these estimates might be used 37

References for appendix 38

S1. Summary statistics

Table S1. Tuberculosis burden, income group, and GBD super region

Country	High tuberculosis burden	WB income group	GBD super region
India	Yes	LM	South Asia
China	Yes	UM	Southeast Asia, East Asia, and Oceania
Angola	Yes	LM	Sub-Saharan Africa
Bangladesh	Yes	LM	South Asia
Brazil	Yes	UM	Latin America and Caribbean
Cambodia	Yes	LM	Southeast Asia, East Asia, and Oceania
Central African Republic	Yes	L	Sub-Saharan Africa
Congo	Yes	LM	Sub-Saharan Africa
Democratic Republic of the Congo	Yes	L	Sub-Saharan Africa
Ethiopia	Yes	L	Sub-Saharan Africa
Indonesia	Yes	LM	Southeast Asia, East Asia, and Oceania
Kenya	Yes	LM	Sub-Saharan Africa
Lesotho	Yes	LM	Sub-Saharan Africa
Liberia	Yes	L	Sub-Saharan Africa
Mozambique	Yes	L	Sub-Saharan Africa
Myanmar	Yes	LM	Southeast Asia, East Asia, and Oceania
Namibia	Yes	UM	Sub-Saharan Africa
Nigeria	Yes	LM	Sub-Saharan Africa
North Korea	Yes	L	Southeast Asia, East Asia, and Oceania
Pakistan	Yes	LM	South Asia
Papua New Guinea	Yes	LM	Southeast Asia, East Asia, and Oceania
Philippines	Yes	LM	Southeast Asia, East Asia, and Oceania
Russian Federation	Yes	UM	Central Europe, Eastern Europe, and Central Asia
Sierra Leone	Yes	L	Sub-Saharan Africa
South Africa	Yes	UM	Sub-Saharan Africa
Tanzania	Yes	L	Sub-Saharan Africa
Thailand	Yes	UM	Southeast Asia, East Asia, and Oceania
Vietnam	Yes	LM	Southeast Asia, East Asia, and Oceania
Zambia	Yes	LM	Sub-Saharan Africa
Zimbabwe	Yes	LM	Sub-Saharan Africa
Afghanistan	No	L	North Africa and Middle East
Albania	No	UM	Central Europe, Eastern Europe, and Central Asia
Algeria	No	UM	North Africa and Middle East
American Samoa	No	UM	Southeast Asia, East Asia, and Oceania
Argentina	No	UM	High-income

Armenia	No	UM	Central Europe, Eastern Europe, and Central Asia
Azerbaijan	No	UM	Central Europe, Eastern Europe, and Central Asia
Belarus	No	UM	Central Europe, Eastern Europe, and Central Asia
Belize	No	UM	Latin America and Caribbean
Benin	No	L	Sub-Saharan Africa
Bhutan	No	LM	South Asia
Bolivia	No	LM	Latin America and Caribbean
Bosnia and Herzegovina	No	UM	Central Europe, Eastern Europe, and Central Asia
Botswana	No	UM	Sub-Saharan Africa
Bulgaria	No	UM	Central Europe, Eastern Europe, and Central Asia
Burkina Faso	No	L	Sub-Saharan Africa
Burundi	No	L	Sub-Saharan Africa
Cameroon	No	LM	Sub-Saharan Africa
Cape Verde	No	LM	Sub-Saharan Africa
Chad	No	L	Sub-Saharan Africa
Colombia	No	UM	Latin America and Caribbean
Comoros	No	LM	Sub-Saharan Africa
Costa Rica	No	UM	Latin America and Caribbean
Cote d'Ivoire	No	LM	Sub-Saharan Africa
Cuba	No	UM	Latin America and Caribbean
Djibouti	No	LM	Sub-Saharan Africa
Dominica	No	UM	Latin America and Caribbean
Dominican Republic	No	UM	Latin America and Caribbean
Ecuador	No	UM	Latin America and Caribbean
Egypt	No	LM	North Africa and Middle East
El Salvador	No	LM	Latin America and Caribbean
Equatorial Guinea	No	UM	Sub-Saharan Africa
Eritrea	No	L	Sub-Saharan Africa
Federated States of Micronesia	No	LM	Southeast Asia, East Asia, and Oceania
Fiji	No	UM	Southeast Asia, East Asia, and Oceania
Gabon	No	UM	Sub-Saharan Africa
Georgia	No	UM	Central Europe, Eastern Europe, and Central Asia
Ghana	No	LM	Sub-Saharan Africa
Grenada	No	UM	Latin America and Caribbean
Guatemala	No	UM	Latin America and Caribbean
Guinea	No	L	Sub-Saharan Africa
Guinea-Bissau	No	L	Sub-Saharan Africa
Guyana	No	UM	Latin America and Caribbean
Haiti	No	L	Latin America and Caribbean
Honduras	No	LM	Latin America and Caribbean
Iran	No	UM	North Africa and Middle East

Iraq	No	UM	North Africa and Middle East
Jamaica	No	UM	Latin America and Caribbean
Jordan	No	UM	North Africa and Middle East
Kazakhstan	No	UM	Central Europe, Eastern Europe, and Central Asia
Kiribati	No	LM	Southeast Asia, East Asia, and Oceania
Kyrgyzstan	No	LM	Central Europe, Eastern Europe, and Central Asia
Laos	No	LM	Southeast Asia, East Asia, and Oceania
Lebanon	No	UM	North Africa and Middle East
Libya	No	UM	North Africa and Middle East
Macedonia	No	UM	Central Europe, Eastern Europe, and Central Asia
Madagascar	No	L	Sub-Saharan Africa
Malawi	No	L	Sub-Saharan Africa
Malaysia	No	UM	Southeast Asia, East Asia, and Oceania
Maldives	No	UM	Southeast Asia, East Asia, and Oceania
Mali	No	L	Sub-Saharan Africa
Marshall Islands	No	UM	Southeast Asia, East Asia, and Oceania
Mauritania	No	LM	Sub-Saharan Africa
Mauritius	No	UM	Southeast Asia, East Asia, and Oceania
Mexico	No	UM	Latin America and Caribbean
Moldova	No	LM	Central Europe, Eastern Europe, and Central Asia
Mongolia	No	LM	Central Europe, Eastern Europe, and Central Asia
Montenegro	No	UM	Central Europe, Eastern Europe, and Central Asia
Morocco	No	LM	North Africa and Middle East
Nepal	No	L	South Asia
Nicaragua	No	LM	Latin America and Caribbean
Niger	No	L	Sub-Saharan Africa
Palestine	No	LM	North Africa and Middle East
Paraguay	No	UM	Latin America and Caribbean
Peru	No	UM	Latin America and Caribbean
Romania	No	UM	Central Europe, Eastern Europe, and Central Asia
Rwanda	No	L	Sub-Saharan Africa
Saint Lucia	No	UM	Latin America and Caribbean
Saint Vincent and the Grenadines	No	UM	Latin America and Caribbean
Samoa	No	UM	Southeast Asia, East Asia, and Oceania
Sao Tome and Principe	No	LM	Sub-Saharan Africa
Senegal	No	LM	Sub-Saharan Africa
Serbia	No	UM	Central Europe, Eastern Europe, and Central Asia
Solomon Islands	No	LM	Southeast Asia, East Asia, and Oceania

Somalia	No	L	Sub-Saharan Africa
South Sudan	No	L	Sub-Saharan Africa
Sri Lanka	No	UM	Southeast Asia, East Asia, and Oceania
Sudan	No	LM	North Africa and Middle East
Suriname	No	UM	Latin America and Caribbean
eSwatini	No	LM	Sub-Saharan Africa
Syria	No	L	North Africa and Middle East
Tajikistan	No	L	Central Europe, Eastern Europe, and Central Asia
The Gambia	No	L	Sub-Saharan Africa
Timor-Leste	No	LM	Southeast Asia, East Asia, and Oceania
Togo	No	L	Sub-Saharan Africa
Tonga	No	UM	Southeast Asia, East Asia, and Oceania
Tunisia	No	LM	North Africa and Middle East
Turkey	No	UM	North Africa and Middle East
Turkmenistan	No	UM	Central Europe, Eastern Europe, and Central Asia
Uganda	No	L	Sub-Saharan Africa
Ukraine	No	LM	Central Europe, Eastern Europe, and Central Asia
Uzbekistan	No	LM	Central Europe, Eastern Europe, and Central Asia
Vanuatu	No	LM	Southeast Asia, East Asia, and Oceania
Venezuela	No	UM	Latin America and Caribbean
Yemen	No	L	North Africa and Middle East

Table S2. Number of extracted tabulated data points by year

Input data	2000	2001	2002	2003	2004	2005	2006	2007	2008	2009	2010	2011	2012	2013	2014	2015	2016	2017
Government health spending on tuberculosis	2	7	5	5	32	48	48	59	61	30	11	18	42	60	57	45	45	4
Prepaid private health spending on tuberculosis	0	0	0	0	0	0	0	0	0	1	3	5	10	8	7	8	4	0
Out-of-pocket health spending on tuberculosis	0	0	1	0	0	1	1	2	0	1	3	5	14	12	12	8	4	0
Government health spending	135	135	135	135	135	135	135	135	135	135	135	135	135	135	135	135	135	135
Out-of-pocket health spending	135	135	135	135	135	135	135	135	135	135	135	135	135	135	135	135	135	135
Domestic National Tuberculosis Programme spending	0	0	1	66	65	70	74	85	79	78	79	81	86	89	84	88	89	87
TB case notifications	0	0	115	115	116	118	118	118	118	118	118	119	119	119	119	119	119	119
MDR-TB cases starting treatment	0	0	116	116	116	118	118	118	118	118	118	118	119	119	119	119	119	119
Health facility visits per case - drug-susceptible TB	0	0	116	116	116	118	118	118	118	118	118	118	119	119	119	119	119	119
Health facility visits per case - drug-resistant TB	0	0	116	116	116	118	118	118	118	118	118	118	119	119	119	119	119	119
Percent of cases admitted - drug-susceptible TB	0	0	115	115	115	117	117	117	117	117	117	117	118	118	118	118	118	118
Percent of cases admitted - drug-resistant TB	0	0	116	116	116	118	118	118	118	118	118	118	119	119	119	119	119	119
Cost of an outpatient visit	0	0	0	0	0	0	0	0	0	0	0	0	0	0	0	0	0	119
Cost of an inpatient bedday	0	0	0	0	0	0	0	0	0	0	0	0	0	0	0	0	0	119
Incident cases - drug-susceptible TB	135	135	135	135	135	135	135	135	135	135	135	135	135	135	135	135	135	135
Incident cases - drug-resistant TB	135	135	135	135	135	135	135	135	135	135	135	135	135	135	135	135	135	135
Government health spending on TB	2	7	5	5	32	48	48	59	61	30	11	18	42	60	57	45	45	4
Prepaid private health spending on TB	0	0	0	0	0	0	0	0	0	1	3	5	10	8	7	8	4	0

Out-of-pocket health spending on TB	0	0	1	0	0	1	1	2	0	1	3	5	14	12	12	8	4	0
Domestic National Tuberculosis Programme spending	0	0	1	66	65	70	74	85	79	78	79	81	86	89	84	88	89	87
Total direct medical costs; TB patient perspective	1	2	1	2	0	0	1	3	3	0	5	0	1	1	1	0	2	2

Table S3. Summary statistics by input data units and sources

Input data	Source	Unit	Observations	Mean	Median	Standard deviation
Government health spending on TB	National Health Accounts	Millions of 2019 US Dollars	111	11	2	34
Prepaid private health spending on TB	National Health Accounts	Millions of 2019 US Dollars	46	1	0	3
Out-of-pocket health spending on TB	National Health Accounts	Millions of 2019 US Dollars	64	44	1	126
Government health spending on TB	Global Fund Proposals	Millions of 2019 US Dollars	261	16	2	73
Government health spending on TB	Global Fund Concept Notes	Millions of 2019 US Dollars	34	10	3	16
Government health spending on TB	National TB Programme reports	Millions of 2019 US Dollars	9	47	6	51
Government health spending on TB	Ministry of Health reports	Millions of 2019 US Dollars	14	1	1	0
Government health spending on TB	Global Fund Funding Landscape Documents	Millions of 2019 US Dollars	7	2	3	2
Government health spending on TB	WHO Global Health Expenditure Database	Millions of 2019 US Dollars	139	11	2	32
Government health spending	IHME	Millions of 2019 US Dollars	2430	3558	252	17968
Out-of-pocket health spending	IHME	Millions of 2019 US Dollars	2430	2951	336	13384
Domestic National TB Programme spending	WHO	Millions of 2019 US Dollars	1201	21	1	129
TB case notifications	WHO	Thousands	1887	49	8	167
MDR-TB cases starting treatment	WHO	Thousands	1888	1	0	2
Health facility visits per case - drug-susceptible TB	WHO	Visits per case	1888	65	64	72
Health facility visits per case - drug-resistant TB	WHO	Visits per case	1888	190	24	248
Percent of cases admitted - drug-susceptible TB	WHO	Percent	1872	28	10	33
Percent of cases admitted - drug-resistant TB	WHO	Percent	1888	62	90	42
Cost of an outpatient visit	WHO CHOICE	2019 US Dollars	119	36	4	325
Cost of an inpatient bedday	WHO CHOICE	2019 US Dollars	119	173	25	1425
Incident cases - drug-susceptible TB	IHME	Thousands	2430	74	10	266
Incident cases - drug-resistant TB	IHME	Thousands	2430	4	0	17
Total direct medical costs; TB patient perspective	Peer-reviewed Literature	2019 US Dollars	25	2059	60	9630

Table S4. Total direct medical costs for tuberculosis patients extracted from peer-reviewed literature

Country	Survey Year	Drug Resistance	Direct medical costs excluding tuberculosis drugs (2019 USD)	Study
Ethiopia	2013	MDR	139.02	Collins, 2014
India	2016	DS	0	Fuady, 2018
India	2016	MDR	9.6	Fuady, 2018
Kenya	2017	DS	9.57	
Kenya	2017	MDR	8.09	
Latvia	2010-2011	MDR	48274.69	Miller, 2013
Mongolia	2018	DS	221.03	Oh, 2018
Mongolia	2018	MDR	450.28	Oh, 2018
South Korea	1999-2004	DS	679.35	Kang, 2006
South Korea	1999-2004	MDR	18509.21	Kang, 2006
Vietnam	2018	DS	137.28	Oh, 2018
Vietnam	2018	MDR	803.18	Oh, 2018
Benin	2008-2009	DS	26.75	Laokri, 2014
Brazil	2007-2008	DS	106.37	Steffen, 2010
China	2002-2005	DS	395.23	Jackson, 2006
China	2012	DS	765.56	Zhou, 2016
Dominican Republic	2010	DS	14.87	Mauch, 2013
Ecuador	2007	DS	385.83	Rouzier, 2010
Ecuador	2007	MDR	410.31	Rouzier, 2010
Ghana	2010	DS	20.81	Mauch, 2013
Haiti	2003	DS	328.45	Jacquet, 2006
India	2000	DS	0.36	Muniyandi, 2005
Malaysia	2010-2011	DS	30.49	Atif, 2014
Nepal	2001-2002	DS	21.1	Karki, 2007
Nigeria	2008	DS	18.74	Umar, 2012
Tajikistan	2006-2007	DS	60.37	Aye, 2010
Thailand	2014-2015	DS	59.72	Tanvejsilp, 2018
Ukraine	2001	DS	92.21	Vassal, 2008
Ukraine	2003	DS	77.11	Vassal, 2008
Vietnam	2010	DS	28.9	Mauch, 2013
Yemen	2008-2009	DS	201.96	Othman, 2010

Table S5. Dominating financing source among 30 high burden countries

30 High Burden Countries				
**Country**	**ISO3**	**World Bank income group**	**Dominating financing source**	**Percent of total health spending on TB from primary source**
Angola	AGO	LM	Government	60.0 (49.5-69.0)
Bangladesh	BGD	LM	Development assistance for health	53.9 (45.1-62.3)
Brazil	BRA	UM	Government	91.5 (83.8-96.3)
Cambodia	KHM	LM	Government	67.8 (63.8-71.4)
Central African Republic	CAF	L	Development assistance for health	65.2 (60.4-69.4)
China	CHN	UM	Government	79.3 (65.6-89.5)
Congo	COG	LM	Government	45.3 (35.6-55.1)
Democratic People's Republic of Korea	PRK	L	Government	95.5 (91.1-98.0)
Democratic Republic of the Congo	COD	L	Out-of-pocket	52.5 (41.9-62.2)
Ethiopia	ETH	L	Government	41.5 (33.9-49.5)
India	IND	LM	Government	47.7 (33.7-61.2)
Indonesia	IDN	LM	Government	56.8 (45.8-66.9)
Kenya	KEN	LM	Development assistance for health	44.1 (37.0-50.9)
Lesotho	LSO	LM	Development assistance for health	70.1 (66.4-73.7)
Liberia	LBR	L	Government	33.1 (23.6-43.3)
Mozambique	MOZ	L	Development assistance for health	40.1 (33.9-46.6)
Myanmar	MMR	LM	Development assistance for health	58.5 (46.8-67.7)
Namibia	NAM	UM	Government	75.7 (71.5-79.5)
Nigeria	NGA	LM	Out-of-pocket	51.6 (35.1-68.9)
Pakistan	PAK	LM	Out-of-pocket	43.8 (28.5-58.7)
Papua New Guinea	PNG	LM	Government	57.5 (51.3-63.9)
Philippines	PHL	LM	Government	72.9 (67.0-77.8)
Russian Federation	RUS	UM	Government	90.0 (83.3-94.8)
Sierra Leone	SLE	L	Government	71.6 (67.4-75.0)
South Africa	ZAF	UM	Government	67.3 (59.3-74.4)
Thailand	THA	UM	Government	64.5 (53.5-73.9)
United Republic of Tanzania	TZA	L	Out-of-pocket	38.9 (31.5-46.7)
Viet Nam	VNM	LM	Government	60.1 (48.1-69.8)
Zambia	ZMB	LM	Development assistance for health	56.3 (53.0-59.3)
Zimbabwe	ZWE	LM	Out-of-pocket	37.0 (29.6-45.4)

S2. Estimating tuberculosis domestic spending

S2.1. Modeling methods: ST-GPR

In order to produce a complete time series of estimates that are comparable among countries and over time, we used spatiotemporal Gaussian process regression (ST-GPR) to estimate government, out-of-pocket, and prepaid private spending on tuberculosis (TB). Overall, we used primary data when available, and relied on ST-GPR to fill missingness when there was no data. ST-GPR is a modeling framework developed by IHME, used extensively in the Global Burden of Disease Study (GBD) and resource tracking. ST-GPR involves in three main steps: 1) Linear modeling with covariates to produce a complete time-series, 2) spatiotemporal smoothing, and 3) Gaussian process regression (GPR). The GPR model utilizes Bayesian inference to update predicted health spending as a posterior in Bayes rule by combining data and a prior probability distribution over parameters in mean, covariance function, and the regression model. For each model, we identified covariates based upon a 10-fold cross validation with out-of-sample tests to avoid over-fit or under-fit, and then selected the model with the smallest root mean square error.

In ST-GPR, there is the option to smooth across countries, which is more applicable for estimating disease burden. In financial estimation, we often do not often smooth across space. Health financing variables vary dramatically across countries because health systems are so distinct. We rely relatively more on the underlying data, the linear fit from the first stage of the model, and smoothing across time. In addition, to reflect reforms and fluctuations in National Tuberculosis Programme (NTP) investment over time, we set a low GPR scale to lower the correlation between data points through time. Finally, we set a low amplitude to narrow variance for countries with good primary data (e.g., country-year>5). Table S5 below shows the parameters used for ST-GPR models run in this analysis:

Table S5. **ST-GPR parameter settings for domestic health spending on tuberculosis models**

Model	Lambda	Omega	Zeta	GPR amp factor	GPR scale
Government health spending on tuberculosis	0.5	0.2	0.001	1	1
Out-of-pocket health spending on tuberculosis	0.5	0.2	0.001	1	1
Prepaid private health spending on tuberculosis	0.5	0.2	0.001	1	5

S2.2. Data sources, assumptions, and estimates

We assume all pre-diagnosis outpatient spending is sourced with the same proportions of government, out-of-pocket, and prepaid private spending as average patients in health systems. For notified cases, we assume diagnostic spending and TB medicine spending are covered by NTP. For non-notified cases, we assume that patients do not access regulated diagnostics, and that TB medicines are sourced in the same way as average patients in health systems. We assume that outpatient and inpatient care are fully covered by government for notified TB cases. For non-notified cases, we assume inpatient and outpatient spending is sourced in the same way as average patients in health systems. As such, the equation below illustrates the components of domestic spending on TB:

$$\begin{aligned} &TB\ domestic\ spending \\ &\quad = Pre\text{-}diagnosis\ outpatient\ spending_{incident\ cases} \\ &\quad + Diagnostic\ spending_{notified|non\text{-}notified} + Drug\ spending_{notified|non\text{-}notified} \\ &\quad + Spending\ on\ healthcare\ utilization\ in\ treatment_{notified|non\text{-}notified} \end{aligned}$$

To estimate government health spending, out-of-pocket health spending, and prepaid private health spending on TB, we created estimates using costs and volume of TB health services. The equations for our cost-volume estimates for each source are as follows:

$$\begin{aligned} &Government_{TB} \\ &\quad = Government_{pre\text{-}treatment} + Government_{notified\ cases} \\ &\quad + Government_{non\text{-}notified\ cases} \end{aligned}$$

$$Out\text{-}of\text{-}pocket_{TB} = Out\text{-}of\text{-}pocket_{pre\text{-}treatment} + Out\text{-}of\text{-}pocket_{non\text{-}notified\ cases}$$

$$Prepaid\ private_{TB} = Prepaid\ private_{pre\text{-}treatment} + Prepaid\ private_{non\text{-}notified\ cases}$$

S2.2.1. Pre-treatment

All TB patients are grouped into two types: drug susceptible patients starting first-line TB treatment and drug resistant patients starting second-line TB treatment. We used different healthcare utilisation quantities and unit cost per patient for drug susceptible- and drug-resistant-TB patients to estimate health system spending. Drug resistant TB includes: RR-TB (Rifampicin-resistant), MDR-TB (Rifampicin- and isoniazid-resistant), and XDR-TB (Extensively drug-resistant TB (XDR TB) is a rare type of multidrug-resistant tuberculosis (MDR TB) that is resistant to isoniazid and rifampin, plus any fluoroquinolone (FQ) and at least one of three injectable second-line drugs (i.e., amikacin, kanamycin, or capreomycin). In this study, we refer all drug resistant tuberculosis as MDR-TB.

We assumed pre-treatment spending was primarily on outpatient care. Pre-treatment visits per patient data were taken from World Health Organization (WHO) patient cost surveys as well as peer-reviewed literature[1,2]. As a function of our assumption that all incident cases sought treatment, we assumed that all incident cases had the same utilisation of pre-treatment visits. We assumed that notified and non-notified cases had the same utilisation of outpatient visits before receiving treatment. To estimate uncertainty around pre-treatment visits, we created 1,000 draws using the 12 data points for each of drug-susceptible (DS) and MDR TB. As such, the data we used was not country-specific. Per the conceptual framework, we assume the spending on pre-treatment care is sourced similarly to the health sector.

To get pre-treatment spending for government health spending, out-of-pocket health spending, and prepaid private health spending, we used the equations below:

$$
\begin{aligned}
&Government_{pre\text{-}treatment} \\
&\quad = incident\ cases * \ pre\text{-}treatment\ visits\ per\ patient_{DS|MDR} \\
&\quad * outpatient\ visit\ unit\ cost * \frac{Government\ outpatient\ spending}{Domestic\ outpatient\ spending}
\end{aligned}
$$

$$
\begin{aligned}
&Out\text{-}of\text{-}pocket_{pre\text{-}treatment} \\
&\quad = incident\ cases * \ pre\text{-}treatment\ visits\ per\ patient_{DS|MDR} \\
&\quad * outpatient\ visit\ unit\ cost * \frac{Out\text{-}of\text{-}pocket\ outpatient\ spending}{Domestic\ outpatient\ spending}
\end{aligned}
$$

$$Prepaid\ private_{pre\text{-}treatment} = incident\ cases * \ pre\text{-}treatment\ visits\ per\ patient_{DS|MDR} * outpatient\ visit\ unit\ cost * \frac{Prepaid\ private\ outpatient\ spending}{Domestic\ outpatient\ spending}$$

To ensure that the medical costs reflected the health system perspective, we adjusted the unit costs with the fractions of outpatient spending sourced by government, out-of-pocket, and prepaid private health spending. We modelled 630, 493, and 486 country-year data points from National Health Accounts (NHAs) respectively, logit-transformed, with ST-GPR. Each model used natural log-transformed lag-distributed income (LDI) per capita from GBD,[3] random effects on country, and the logit-transformed fraction of total health spending per each source ($\frac{Government\ health\ spending}{Domestic\ health\ spending}$, $\frac{Out\text{-}of\text{-}pocket\ health\ spending}{Domestic\ health\ spending}$, and $\frac{Prepaid\ private\ health\ spending}{Domestic\ health\ spending}$) from the GBD Health Financing Collaborator Network[4] as covariates.

S2.2.2. Notified cases

We assumed that all spending on notified cases was sourced by the government. While we found evidence that indicated that some patients who were treated through National Tuberculosis Programmes incurred out-of-pocket costs, the evidence was limited and the costs were often closer to 0 than to the pattern of out-of-pocket spending in the health sector as a whole. See Table S5 for studies we reviewed on out-of-pocket expenditures incurred by patients in the NTP setting[1, 2,5–9,10–12, 13–21].

Table S6. Out-of-pocket costs incurred by patients treated in a National Tuberculosis Programme setting from peer-reviewed literature

Study	Coun try	Survey Year	Drug Resistance	Total Direct Medical Costs During Treatment (2019 USD)	Measu	Notes
Anathakrishnan, 2012	India	2007	DS	0.6	mean	
Aspler, 2008	Zambia	2006	DS	1.42	median	
Asres, 2018	Ethiopi a	2015	DS	$ -	median	
Asres, 2018	Ethiopi a	2015	DS	4.62	mean	
Fuady, 2018	Indone sia	2016	DS	$ -	median	
Fuady, 2018	Indone sia	2016	MDR	15.41	median	
Jackson, 2006	China	2002-2005	DS	375.39	mean	
Jacquet, 2006	Haiti	2003	DS	351.62	mean	
Jacquet, 2006	Haiti	2003	DS	872.92	mean	
John, 2009	India	2007	DS	0.03	mean	
Laokri, 2014	Benin	2008	DS	2.98	median	
Mahendradhata, 2010	Indone sia	2004-2005	DS	3.95	median	
Collins, 2014	Ethiopi a	2013	MDR	46.59	median	
Mauch, 2013	Domini can Republ ic	2010	DS	7.58	median	
Mauch, 2013	Ghana	2010	DS	15.12	median	
Mauch, 2013	Ghana	2010	DS	81.46	mean	
Mauch, 2013	Vietna m	2010	DS	17.61	median	
Muniyandi, 2005	India	2000	DS	$ -	median	
Othman, 2010	Yemen	2008-2009	DS	62.93	mean	Pulmonary
Othman, 2010	Yemen	2008-2009	DS	71.82	mean	Extra-pulmonary
Pantoja, 2009	India	2005	DS	21.36	mean	
Pedrazzoli, 2018	Ghana	2016	DS	93.36	median	
Pedrazzoli, 2018	Ghana	2016	MDR	38.53	median	
Pichenda, 2012	Cambo dia	2008-2009	DS	0.52	mean	HIV-negative
Pichenda, 2012	Cambo dia	2008-2009	DS	$ -	mean	HIV-positive
Pichenda, 2012	Cambo dia	2008-2009	MDR	$ -	mean	
Prasanna, 2018	India	2016-2017	DS	$ -	median	
Rouzier, 2010	Ecuado r	2007	DS	164.14	mean	
Rouzier, 2010	Ecuado r	2007	MDR	418.61	mean	
Vassal, 2008	Ukrain e	2001	DS	3.54	mean	Mariupol, Ukraine

Vassal, 2008	Ukrain e	2003	DS	2.27	mean	Mariupol, Ukraine
Vassal, 2008	Ukrain e	2001	DS	108.39	mean	Kyiv City, Ukraine
Vassal, 2008	Ukrain e	2003	DS	42.12	mean	Kyiv City, Ukraine
Vassal, 2008	Ukrain e	2003	DS	2615.26	mean	Kyiv City, Ukraine
Vassal, 2008	Ukrain e	2004	DS	1440.34	mean	Kyiv City, Ukraine
Vassal, 2008	Ukrain e	2001	DS	2105.24	mean	Mariupol, Ukraine
Vassal, 2008	Ukrain e	2003	DS	1417.7	mean	Mariupol, Ukraine
Xu, 2006	China	2002	DS	23.97	mean	Jianhu province
Xu, 2006	China	2002	DS	38.89	median	Jianhu province
Xu, 2006	China	2002	DS	187.53	mean	Funing province
Xu, 2006	China	2002	DS	151.04	median	Funing province
Yuan, 2017	China		DS	153.34	median	
Zhou, 2016	China	2012	DS	1004.23	mean	
Zhou, 2019	China	2014	DS	227.27	median	

The equation below is the simplest form of calculating $Government_{notified\ cases}$:

$$Government_{notified\ cases} = National\ Tuberculosis\ Program\ spending + Government\ patient\ care_{notified\ cases}$$

We utilized $National\ Tuberculosis\ Program\ spending$ expenditure data reported to the WHO[22]. To fill any missing data, we used ST-GPR to model the reported NTP spending as a logit-transformed fraction of total government health expenditure from the GBD Health Financing Collaborator Network[4] with the following covariates: MDR patients starting treatment through NTP from the WHO Global TB Database[22] and the logit-transformed proportion of total health expenditure sourced by the government from the GBD Health Financing Collaborator Network[4], with random effects on country, region, and GBD super region. The expenditures reported by National Tuberculosis Programmes typically include medication, diagnostics, staff, and other program expenditures.

$Government\ patient\ care_{notified\ cases}$ is comprised of inpatient and outpatient spending for patients that are notified to the NTP. We assume that all drug, diagnostic, and patient tracing costs are borne by the NTP for notified cases. The equation below describes how $Government\ patient\ care_{notified\ cases}$ is estimated:

$$Government\ patient\ care_{notified\ cases} = outpatient\ spending\ on\ notified\ cases_{DS|MDR} + inpatient\ spending\ on\ notified\ cases_{DS|MDR}$$

In the above equation and all following equations, the $_{DS|MDR}$ subscript indicates a variable that has unique values for both drug-susceptible and drug-resistant TB.

The equations below illustrate how each of the components of $Government\ patient\ care_{DS|MDR}$ are calculated:

$$Outpatient\ spending\ on\ notified\ cases_{DS|MDR} = notified\ cases_{DS|MDR} * outpatient\ visit\ unit\ cost * average\ visits\ per\ notified\ case_{DS|MDR}$$

$$Inpatient\ spending\ on\ notified\ cases_{DS|MDR} = notified\ cases_{DS|MDR} * bed\ day\ unit\ cost_{DS|MDR} * average\ inpatient\ duration\ per\ case_{DS|MDR} * \frac{inpatient\ cases}{notified\ cases}_{DS|MDR}$$

$Notified\ cases_{DS|MDR}$ was sourced from the WHO Global TB Database.[22] We used ST-GPR to fill any missing data points for these utilisation variables and create a complete time series of estimates.

All utilisation variables - $average\ visits\ per\ notified\ case_{DS|MDR}$, $average\ inpatient\ duration\ per\ case_{DS|MDR}$, and $\frac{inpatient\ cases}{notified\ cases}_{DS|MDR}$ - were also downloaded from the WHO Global TB Database[22] and modelled with ST-GPR. Unit costs - $outpatient\ visit\ unit\ cost$ and

$bed\ day\ unit\ cost_{DS|MDR}$ – were sourced from WHO CHOICE[23] and modelled in ST-GPR using outpatient visit and inpatient admission unit costs from Moses et al.[24] as covariates respectively.

We used data on inpatient and outpatient spending for tuberculosis sourced from NHAs to fill in $outpatient\ spending\ on\ notified\ cases_{DS|MDR}$ and $inpatient\ spending\ on\ notified\ cases_{DS|MDR}$. We multiplied inpatient spending and outpatient spending on TB from the NHAs by the fraction of $Government\ patient\ care_{notified\ cases}$ on drug-susceptible and drug-resistant TB from our estimates using cost and volume variables to get inpatient and outpatient spending specific to drug-susceptible and drug-resistant TB. To create a full time series for the data from NHAs, we extrapolated the first and last data points in the time series using the annual rate of change for our estimates using the cost and volume components. To fill any missing country-years between the first and last points in the time series, we used linear interpolation.

S2.2.3. Non-notified cases

$Non\text{-}notified\ cases_{DS|MDR}$ can be quantified as $incident\ cases_{DS|MDR} -\ notified\ cases_{DS|MDR}$. Per Table 1 in the manuscript, we assume that inpatient and outpatient care for non-notified cases is sourced similarly to the health system as a whole. We do not account for any spending on drugs sourced by the government through social insurance for non-notified cases, as we assume that government spending on drugs for TB is accounted for in NTP spending.

As such, the equations below illustrate how we calculated spending on non-notified cases by source:

$$\begin{aligned} Government_{non\text{-}notified} &= Government\ outpatient\ spending_{non\text{-}notified\ cases} \\ &+ Government\ inpatient\ spending_{non\text{-}notified\ cases} \end{aligned}$$

$$\begin{aligned} Out\text{-}of\text{-}pocket_{non\text{-}notified} &= Out\text{-}of\text{-}pocket\ outpatient\ spending_{non\text{-}notified\ cases} \\ &+ Out\text{-}of\text{-}pocket\ intpatient\ spending_{non\text{-}notified\ cases} \\ &+ Out\text{-}of\text{-}pocket\ drug\ spending_{non\text{-}notified\ cases} \end{aligned}$$

$$Prepaid\ private_{non\text{-}notified}$$
$$= Prepaid\ private\ outpatient\ spending_{non\text{-}notified\ cases}$$
$$+ Prepaid\ private\ inpatient\ spending_{non\text{-}notified\ cases}$$
$$+ Prepaid\ private\ drug\ spending_{non\text{-}notified\ cases}$$

Outpatient spending by source is calculated as follows:

$$Government\ outpatient\ spending_{non-notified\ cases}$$
$$= non\text{-}notified\ cases_{DS|MDR} * outpatient\ visit\ unit\ cost$$
$$* average\ visits\ per\ notified\ case_{DS|MDR} * \frac{Government\ outpatient\ spending}{Domestic\ outpatient\ spending}$$

$$Out\text{-}of\text{-}pocket\ outpatient\ spending_{non\text{-}notified\ cases}$$
$$= non\text{-}notified\ cases_{DS|MDR} * outpatient\ visit\ unit\ cost$$
$$* average\ visits\ per\ notified\ case_{DS|MDR} * \frac{Out\text{-}of\text{-}pocket\ outpatient\ spending}{Domestic\ outpatient\ spending}$$

$$Prepaid\ private\ spending_{non\text{-}notified\ cases}$$
$$= non\text{-}notified\ cases_{DS|MDR} * outpatient\ visit\ unit\ cost$$
$$* average\ visits\ per\ notified\ case_{DS|MDR} * \frac{Prepaid\ private\ outpatient\ spending}{Domestic\ outpatient\ spending}$$

Inpatient spending by source for non-notified cases is calculated as follows:

$$Government\ inpatient\ spending_{non\text{-}notified\ cases}$$
$$= non\text{-}notified\ cases_{DS|MDR} * \ bed\ day\ unit\ cost_{DS|MDR}$$
$$* \ average\ inpatient\ duration\ per\ case\ _{DS|MDR} \ * \frac{inpatient\ cases}{notified\ cases}_{DS|MDR}$$
$$* \frac{Government\ inpatient\ spending}{Domestic\ inpatient\ spending}$$

$$Out\text{-}of\text{-}pocket\ inpatient\ spending_{non\text{-}notified\ cases} = non\text{-}notified\ cases_{DS|MDR} * \ bed\ day\ unit\ cost_{DS|MDR} * \ average\ inpatient\ duration\ per\ case\ _{DS|MDR} * \frac{inpatient\ cases}{notified\ cases}_{DS|MDR} * \ \frac{Out\text{-}of\text{-}pocket\ inpatient\ spending}{Domestic\ inpatient\ spending}$$

$$Prepaid\ private\ inpatient\ spending_{non\text{-}notified\ cases} = non\text{-}notified\ cases_{DS|MDR} * \ bed\ day\ unit\ cost_{DS|MDR} * \ average\ inpatient\ duration\ per\ case\ _{DS|MDR} * \frac{inpatient\ cases}{notified\ cases}_{DS|MDR} * \ \frac{Prepaid\ private\ inpatient\ spending}{Domestic\ inpatient\ spending}$$

All unit cost and utilisation variables for inpatient and outpatient spending for non-notified cases are the same as the ones used for notified cases. We assumed that health care utilisation patterns were the same for notified cases and non-notified cases. Alternative scenarios are addressed in the sensitivity analysis. To ensure that the inpatient medical costs reflected the health system perspective, we adjusted the inpatient unit costs with the fractions of $\frac{Government\ inpatient\ spend}{Domestic\ inpatient\ spend}$, $\frac{Out\text{-}of\text{-}pocket\ inpatient\ spend}{Domestic\ inpatient\ spend}$, and $\frac{Prepaid\ private\ inpatient\ spend}{Domestic\ inpatient\ spend}$. We modelled 625, 491, and 484 country-year data points from NHAs with ST-GPR. Each model used natural log-transformed LDI per capita from GBD[3], random effects on country, and the logit-transformed fraction of total health spending per each source ($\frac{Government\ health\ spending}{Domestic\ health\ spending}$, $\frac{Out\text{-}of\text{-}pocket\ health\ spending}{Domestic\ health\ spending}$, and $\frac{Prepaid\ private\ health\ spending}{Domestic\ health\ spending}$) from the GBD Health Financing Collaborator Network[4] as covariates.

For out-of-pocket and prepaid private spending, we calculated drug spending as follows:

$$Out\text{-}of\text{-}pocket\ drug\ spending_{non\text{-}notified\ cases} = non\text{-}notified\ cases_{DS|MDR} * drug\ cost\ per\ patient\ _{DS|MDR} * \ \frac{Out\text{-}of\text{-}pocket\ medical\ goods\ spending}{Domestic\ medical\ goods\ spending}$$

$$Prepaid\ private\ drug\ spending_{non\text{-}notified\ cases} = non\text{-}notified\ cases_{DS|MDR} * drug\ cost\ per\ patient_{\ DS|MDR} * \frac{Prepaid\ private\ medical\ goods\ spending}{Domestic\ medical\ goods\ spending}$$

$Drug\ cost\ per\ patient_{DS|MDR}$ data was from the WHO Global TB database[22]. This data represents the reported cost per patient for drugs from the NTP perspective, excluding buffer stock. Reported data excluding buffer stock was only reported from 2015 onward. As such, we modelled the natural log-transformed reported costs per patient in ST-GPR, using the average drug cost per patient including buffer stock as a covariate, to fill in missing data.

The average cost per patient including buffer stock was calculated using total expenditure on drugs from NTPs, including buffer stock, and the number of notified cases. We chose to use this covariate because more data was available and the reported cost per patient shared a statistically significant relationship with the calculated cost per patient including buffer stock when tested in natural log space. While more data on drug expenditures including buffer stock was available, the time series was not complete. To fill in missing data, we modelled the natural log-transformed average drug cost per patient including buffer stock in ST-GPR using natural log-transformed LDI per capita from GBD[3] as a covariate.

To adjust $Drug\ cost\ per\ patient_{DS|MDR}$ to reflect the share of drugs costs sourced out-of-pocket and prepaid private, we utilized $\frac{Out\text{-}of\text{-}pocket\ medical\ goods\ spending}{Domestic\ medical\ goods\ spending}$ and $\frac{Prepaid\ medical\ goods\ spending}{Domestic\ medical\ goods\ spending}$ data, with 537 and 505 country-year data points sourced from NHAs respectively. We used data for HC.5 (“Medical goods (non-specified by function”) from the System of Health Accounts (SHA) 2011 framework in place of HC.5.1 (“Pharmaceuticals and other medical non-durables”) because the two codes shared a significant relationship and more data was available for HC.5. $\frac{Out\text{-}of\text{-}pocket\ medical\ goods\ spending}{Domestic\ medical\ goods\ spending}$ and $\frac{Prepaid\ private\ medical\ goods\ spending}{Domestic\ medical\ goods\ spending}$ were logit-transformed and modelled with ST-GPR. Each model used natural log-transformed LDI per capita[3], random effects on country, and the logit-transformed fraction of total health spending per each source ($\frac{Out\text{-}of\text{-}pocket\ health\ spending}{Domestic\ health\ spending}$ and

$\frac{Prepaid\ private\ health\ spending}{Domestic\ health\ spending}$) from the GBD Health Financing Collaborator Network[4] respectively as covariates.

S2.3. Modeling financing sources and functions

S2.3.1. Modeling total government spending on tuberculosis

To estimate a full time series for all years and countries in our study with uncertainty, we modelled $Government_{TB}$ as a share of total government spending from the GBD Health Financing Collaborator Network[4] with ST-GPR. We first considered the covariates in the table below:

Table S7. Covariates considered

Model	Covariates
**Out-of-pocket health spending on TB**	Out-of-pocket health spending, proportion of out-of-pocket/total health spending, LDI per capita, universal health coverage, BCG vaccine coverage proportion, tuberculosis case notifications, multi-drug resistant tuberculosis patients starting treatment
**Government health spending on TB**	NTP spending, NTP spending per capita, case notifications per capita, average cost of an inpatient stay, average cost of an outpatient visit, LDI per capita, total health expenditure per capita, fraction of case notifications that were multi-drug resistant

We sourced all estimates from the GBD 2017 study and the WHO Global TB Database. The first stage linear model of ST-GPR was a mixed-effect model, with the logit-transformed fraction of NTP spending/total government health expenditure, natural log-transformed case notifications and the logit-transformed fraction of case notifications that were multi-drug resistant from WHO,[22] and natural log-transformed LDI per capita as covariates and random effects on country. To detect and reduce the influence of outlier data points, we used the selected model to measure Cook's distance for each data point. We excluded each data point if Cook's distance was greater than 15/n where n is the total number of $Government_{TB}$ data points.

S2.3.2. Modeling total out-of-pocket spending on tuberculosis

For our final model, we logit-transformed $Out\text{-}of\text{-}pocket_{TB}$ as a fraction of total out-of-pocket health spending from the GBD Health Financing Collaborator Network.[4] To select covariates, we first conducted a lasso regression to determine which covariates were least correlated, conditional on other covariates, with the logit-transformed $Out\text{-}of\text{-}pocket_{TB}$ fraction as the dependent variable. Covariates with an estimated coefficient of zero were removed from the set of possible covariates. We then used linear mixed effects regression to estimate all models including all possible combinations of the remaining covariates.

We then selected the 1000 best models with the lowest Akaike information criterion (AIC) and the 1000 best models with the lowest Bayesian information criterion (BIC) values. Finally, we completed a 10-fold cross-validation with out-of-sample predictions on these selected models. We selected the best model based on out-of-sample root mean squared error.

Table S8. Top OOP spending models based on out-of-sample root-mean square error

Model	Covariates	OOS RMSE
1	LDI per capita, universal health coverage, proportion of out-of-pocket/total health spending	0.705278
2	LDI per capita, universal health coverage, BCG vaccine coverage proportion, proportion of out-of-pocket/total health spending	0.705367
3	LDI per capita, universal health coverage, out-of-pocket health spending, proportion of out-of-pocket/total health spending	0.705428
4	LDI per capita, universal health coverage, tuberculosis case notifications, proportion of out-of-pocket/total health spending	0.705434
5	LDI per capita, universal health coverage, tuberculosis case notifications, proportion of out-of-pocket/total health spending	0.705448

We used Cook's distance to detect outliers and excluded any points with a Cook's distance greater than 15/n, where n is the total number of $Out\text{-}of\text{-}pocket_{TB}$ data points.

S2.3.3. Modeling prepaid private spending on tuberculosis

Prepaid private spending includes spending by voluntary insurance (i.e. private and not mandated) and spending from domestic non-governmental organisations. We modelled $Prepaid\ private_{TB}$ as a logit-transformed fraction of total prepaid private health spending from the GBD Health Financing Collaborator Network.[4] For the linear first stage of ST-GPR, we used a mixed-effects model with the logit-transformed proportion of prepaid private/total health spending and natural log-transformed LDI per capita as covariates with random effects on country. We used Cook's distance to detect outliers and

excluded any points with a Cook's distance greater than 15/n, where n is the total number of $Prepaid\ private_{TB}$ data points.

S2.3.4. Modeling spending on tuberculosis by function

The following components are mutually exclusive and exhaustive of domestic spending on tuberculosis:

$$Government_{pre\text{-}treatment\ DS}$$

$$Government_{pre\text{-}treatment\ MDR}$$

$$National\ Tuberculosis\ Program\ spending$$

$$Government\ inpatient\ spending_{notified\ cases\ DS}$$

$$Government\ inpatient\ spending_{notified\ cases\ MDR}$$

$$Government\ outpatient\ spending_{notified\ cases\ DS}$$

$$Government\ outpatient\ spending_{notified\ cases\ MDR}$$

$$Government\ inpatient\ spending_{non\text{-}notified\ cases\ DS}$$

$$Government\ inpatient\ spending_{non\text{-}notified\ cases\ MDR}$$

$$Government\ outpatient\ spending_{non\text{-}notified\ cases\ DS}$$

$$Government\ outpatient\ spending_{non\text{-}notified\ cases\ MDR}$$

$$Out\text{-}of\text{-}pocket_{pre\text{-}treatment\ DS}$$

$$Out\text{-}of\text{-}pocket_{pre\text{-}treatment\ MDR}$$

$$Out\text{-}of\text{-}pocket\ inpatient\ spending_{non\text{-}notified\ cases\ DS}$$

$$Out\text{-}of\text{-}pocket\ inpatient\ spending_{non\text{-}notified\ cases\ MDR}$$

$$Out\text{-}of\text{-}pocket\ outpatient\ spending_{non\text{-}notified\ cases\ DS}$$

$$Out\text{-}of\text{-}pocket\ outpatient\ spending_{non\text{-}notified\ cases\ MDR}$$

$$Out\text{-}of\text{-}pocket\ drug\ spending_{non\text{-}notified\ cases\ DS}$$

$$Out\text{-}of\text{-}pocket\ drug\ spending_{non\text{-}notified\ cases\ MDR}$$

$$Prepaid\ private_{pre\text{-}treatment\ DS}$$

$$Prepaid\ private_{pre\text{-}treatment\ MDR}$$

$$Prepaid\ private\ inpatient\ spending_{non\text{-}notified\ cases\ DS}$$

$$Prepaid\ private\ inpatient\ spending_{non\text{-}notified\ cases\ MDR}$$

$$Prepaid\ private\ outpatient\ spending_{non\text{-}notified\ cases\ DS}$$

$$Prepaid\ private\ outpatient\ spending_{non\text{-}notified\ cases\ MDR}$$

$$Preapid\ private\ drug\ spending_{non\text{-}notified\ cases\ DS}$$

$$Prepaid\ private\ drug\ spending_{non\text{-}notified\ cases\ MDR}$$

To produce final estimates of each component to be able to calculate spending on health functions that scale properly to the source estimates, we calculated each component as a fraction of its corresponding financing source. For example, $Government\ inpatient\ spending_{notified\ cases\ DS}/Government_{TB}$. We then multiplied these proportions by our final estimates by source to get values for each component. To produce estimates by function, we aggregated each component of each function across sources, as illustrated below:

$$\begin{aligned} Inpatient\ spending & \\ = {} & Government\ inpatient\ spending_{notified\ cases\ DS} \\ & + Government\ inpatient\ spending_{notified\ cases\ MDR} \\ & + Government\ inpatient\ spending_{non\text{-}notified\ cases\ DS} \\ & + Government\ inpatient\ spending_{non\text{-}notified\ cases\ MDR} \\ & + Out\text{-}of\text{-}pocket\ inpatient\ spending_{non\text{-}notified\ cases\ DS} \\ & + Out\text{-}of\text{-}pocket\ inpatient\ spending_{non\text{-}notified\ cases\ MDR} \\ & + Prepaid\ private\ spending_{non\text{-}notified\ cases\ DS} \\ & + Prepaid\ private\ spending_{non\text{-}notified\ cases\ MDR} \end{aligned}$$

We produced function estimates for National Tuberculosis Programme spending (inclusive of development assistance and exclusive of drug spending), inpatient spending, outpatient spending, and drug spending (inclusive of both private and NTP drug spending).

Currently, we do not break down NTP spending into any further functions such as diagnostic spending, nor do we estimate spending on immunisation. These are limitations of our current estimates, but we plan to incorporate them into future work.

S3. Currency Conversion

All TB expenditure estimates were made and reported in 2019 United States dollars (USD). Data sources reported expenditure in either nominal local currency units (LCUs) or nominal USD. To convert nominal LCUs to USD, we applied deflators to nominal LCUs to inflate to 2019 LCUs. We then applied exchange rates to produce 2019 USD. When LCUs were not reported, we extracted reported expenditure in nominal USD, applied corresponding nominal exchange rates to produce nominal LCUs, inflated nominal LCUs to 2019 LCUs with deflators, and finally exchanged 2019 LCUs to 2019 USD. All deflators and exchange rates were extracted from the World Bank, International Monetary Fund, Penn World Tables, the United Nations National Accounts and the World Health Organisation, and were imputed to provide a complete series for each of the variables between 1950 and 2019. We then used several models including ordinary least-squares regression and mixed effects models, to complete each source series. More information about the approach to converters and deflators may be found in GBD Health Financing Collaborator Network (2020)[4].

S4. Sensitivity analysis

We conducted sensitivity analyses using different key variables and data sources to test if our models were input dependent.

Model 1 below represents our primary estimates. Model 2 addresses one of our key assumptions, which is that all TB cases seek treatment. In Model 2, we assume that only 75% of non-notified cases sought treatment. Model 3 addresses another of our key assumptions, which is that non-notified cases have similar healthcare utilisation patterns as notified cases. In Model 3, we assume that health care utilisation (health facility visits and hospitalisation percentage) are 50% lower for non-notified cases than notified cases. Finally, Model 4 tests the sensitivity of our estimates to different incidence inputs. In Model 4, we use WHO reported estimates of incidence for TB instead of IHME estimates of TB incidence.

Table S9. Sensitivity analysis model results

	Model 1		Model 2		Model 3		Model 4	
	Mean	CI	Mean	CI	Mean	CI	Mean	CI
**Total spend on tuberculosis in millions of USD**	10941.9	10273.7 to 11753.9	9926.4	9293.9 to 10661.5	8270.9	7703.7 to 8887.5	10729.5	10073.5 to 11549.5
**Total spend on tuberculosis per incident case in USD**	1243	1167.1 to 1335.2	1127.6	1055.8 to 1211.1	939.6	875.1 to 1009.6	1218.9	1144.3 to 1312.0
**Tuberculosis development assistance as fraction of total tuberculosis**	15.8	14.7 to 16.8	17.4	16.2 to 18.5	20.8	19.4 to 22.4	16.1	14.9 to 17.1
**Tuberculosis government as fraction of total tuberculosis**	63.5	59.2 to 66.8	65.5	61.5 to 68.6	59.8	55.9 to 63.1	63.3	59.2 to 66.6
**Tuberculosis out-of-pocket as fraction of total tuberculosis**	18.7	15.2 to 23.6	15.5	12.1 to 20.2	17.4	14.1 to 22.1	18.6	15.0 to 23.3
**Total spend on tuberculosis 2000-2017 annual growth rate**	3.9	3.0 to 4.6	4.5	3.7 to 5.2	4.4	3.6 to 5.2	4	3.1 to 4.8

Despite differences between the mean results across models, the qualitative conclusions drawn from each model remain the same, as illustrated in the figures below:

Figure S1. Tuberculosis spending by source in 135 Low and Middle Income Countries, Model 1

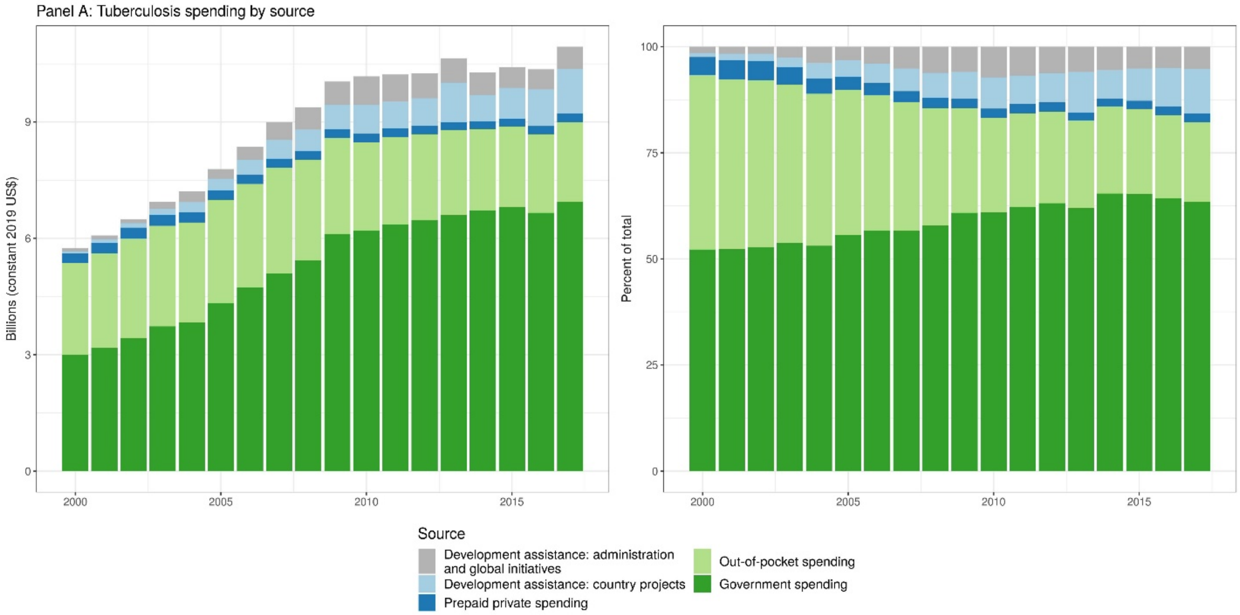

Figure S2. Tuberculosis spending by source in 135 Low and Middle Income Countries, Model 2

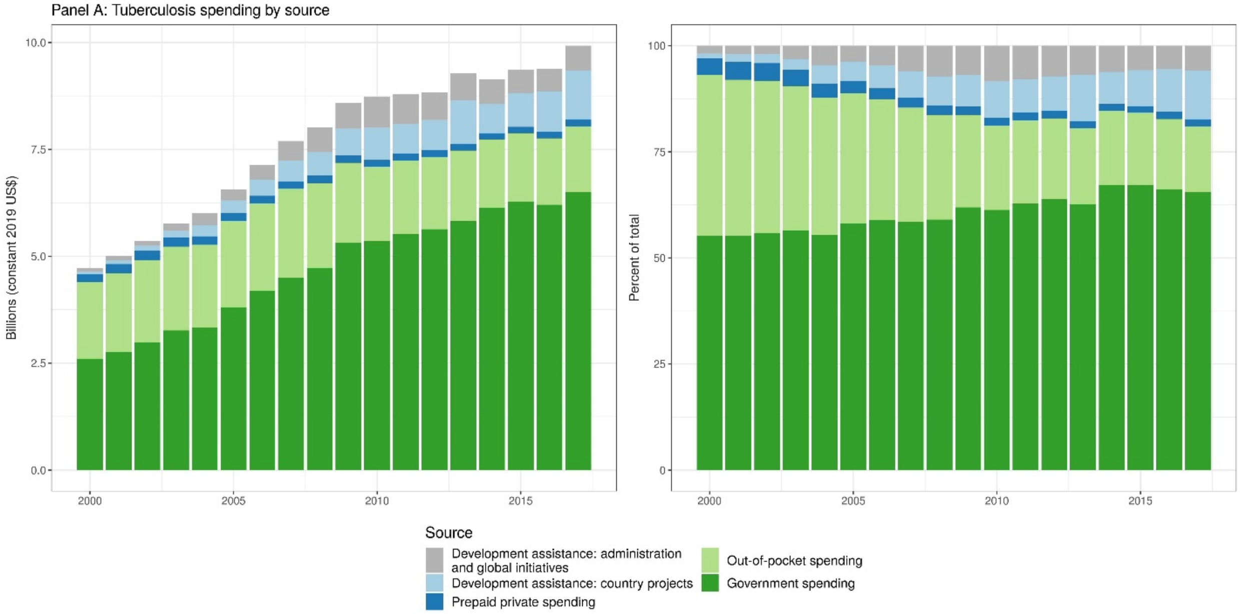

Figure S3. Tuberculosis spending by source in 135 Low and Middle Income Countries, Model 3

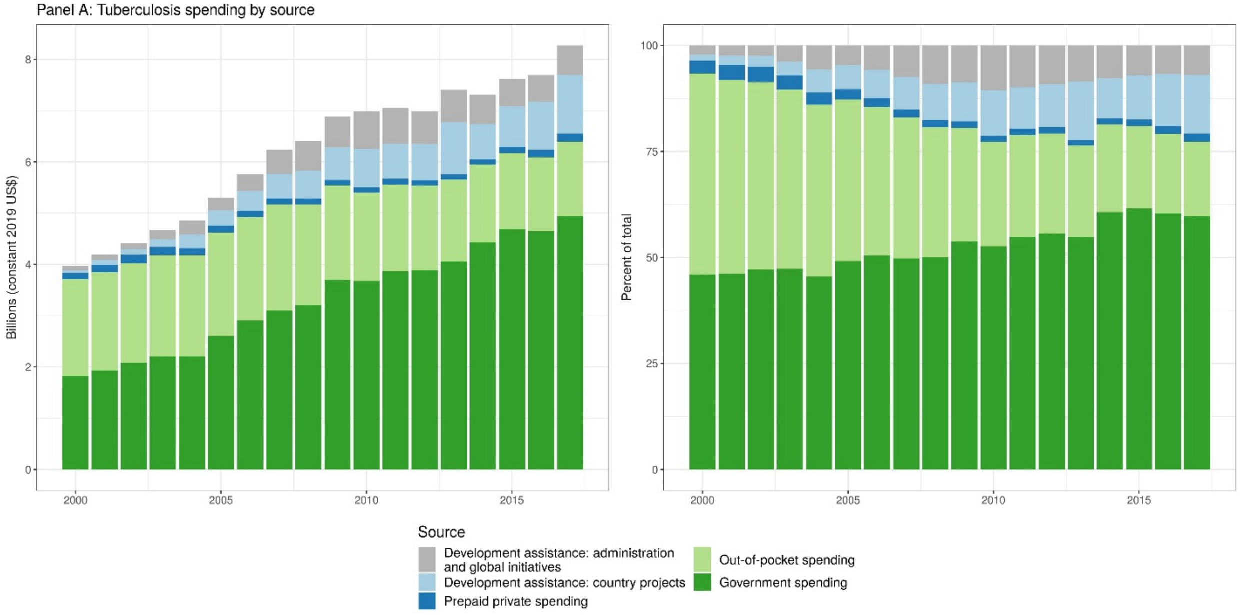

Figure S4. Tuberculosis spending by source in 135 Low and Middle Income Countries, Model 4

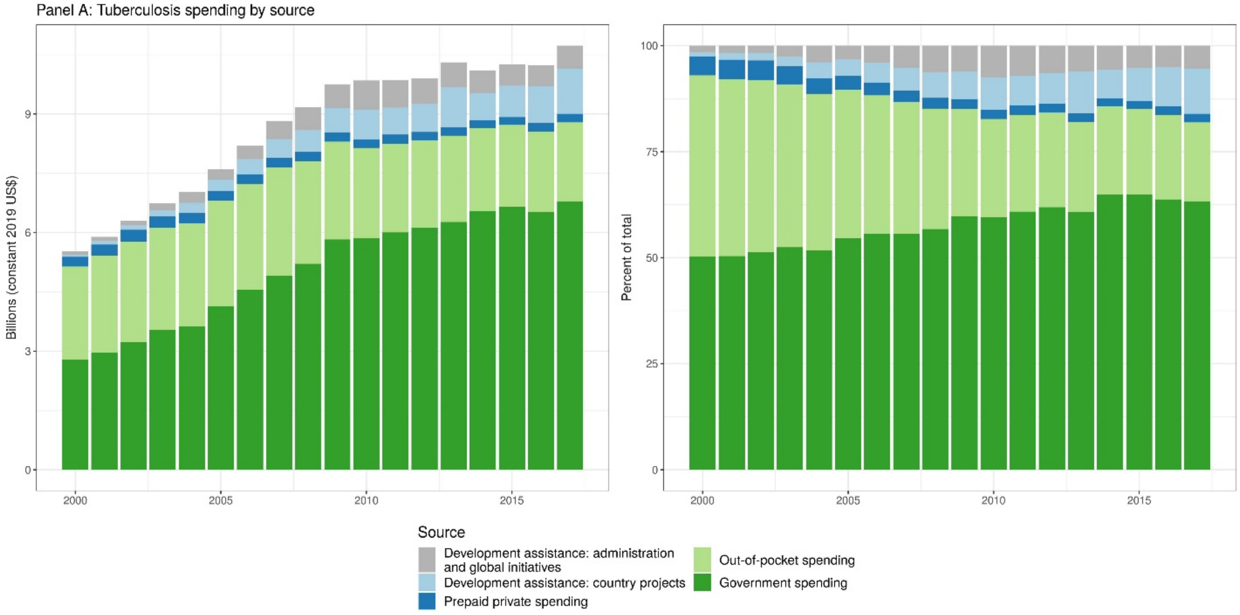

S5. Results in purchasing power parity (PPP) dollars

Table S10. Tuberculosis spending countries in 135 Low and Middle Income Countries, 2017

Location	Total spend on tuberculosis in millions of PPP	Total spend on tuberculosis per incident case in PPP	Tuberculosis DAH in millions of PPP	Tuberculosis government in millions of PPP	Tuberculosis out-of-pocket in millions of PPP	Total spend on tuberculosis as a fraction of total health expenditure
135 low- and middle-income countries	30646.4 (28568.7 to 33246.8)	3013.2 (2808.9 to 3268.9)	13.4 (12.4 to 14.4)	62.8 (57.7 to 66.7)	21.6 (17.2 to 27.5)	4.2 (3.4 to 4.9)
India + China	9219.8 (7359.6 to 11711.4)	2448.4 (1954.4 to 3110.1)	4.7 (3.6 to 5.8)	54.7 (41.5 to 66.1)	37.9 (25.4 to 51.8)	4.2 (2.0 to 6.5)
28 other high-burden countries	12724.4 (11731.8 to 13751.7)	2708.0 (2496.8 to 2926.7)	16.7 (15.4 to 18.0)	64.3 (60.4 to 67.5)	16.8 (13.6 to 21.2)	4.0 (3.2 to 4.8)
Other LMICs	8126.1 (7732.7 to 8592.8)	4762.6 (4532.0 to 5036.1)	12.2 (11.6 to 12.8)	74.6 (72.9 to 76.3)	11.9 (10.5 to 13.4)	4.1 (3.7 to 4.5)
Low-income countries	2407.6 (2310.3 to 2515.6)	1134.4 (1088.5 to 1185.2)	33.6 (32.1 to 35.0)	30.0 (28.2 to 31.8)	31.4 (29.0 to 34.2)	4.7 (4.3 to 5.2)
Lower-middle-income countries	14065.6 (12287.5 to 16418.7)	2267.6 (1981.0 to 2647.0)	16.3 (13.9 to 18.5)	47.3 (39.6 to 53.9)	34.0 (25.7 to 44.0)	5.7 (4.5 to 7.0)
Upper-middle-income countries	13597.0 (12539.7 to 14805.8)	7368.0 (6795.1 to 8023.0)	3.3 (3.0 to 3.6)	87.5 (84.0 to 90.1)	7.9 (5.3 to 11.5)	2.7 (1.6 to 3.7)
Central Europe, Eastern Europe, and Central Asia	9709.0 (8861.5 to 10604.6)	40194.5 (36685.9 to 43902.2)	3.7 (3.4 to 4.1)	87.5 (83.4 to 90.5)	8.2 (5.3 to 12.5)	4.9 (3.9 to 5.9)
Latin America and Caribbean	1126.2 (1001.5 to 1259.6)	5680.2 (5051.3 to 6352.8)	4.4 (3.9 to 4.9)	91.5 (89.9 to 92.8)	3.4 (2.4 to 4.8)	2.3 (1.3 to 3.4)
North Africa and Middle East	1123.0 (1002.0 to 1273.6)	4626.1 (4127.4 to 5246.2)	10.4 (9.1 to 11.6)	79.2 (75.6 to 82.4)	10.0 (7.3 to 13.2)	4.4 (3.2 to 5.5)
South Asia	8277.1 (6560.9 to 10634.4)	2352.6 (1864.8 to 3022.6)	10.3 (7.9 to 12.8)	44.0 (32.2 to 54.7)	43.1 (30.4 to 57.1)	6.2 (4.0 to 8.5)
Southeast Asia, East Asia, and Oceania	4404.2 (3805.1 to 5118.8)	2100.2 (1814.5 to 2441.0)	13.8 (11.8 to 15.9)	70.9 (64.0 to 76.6)	13.4 (8.6 to 19.8)	0.9 (-1.2 to 2.8)
Sub-Saharan Africa	5270.0 (4955.9 to 5696.0)	1364.4 (1283.0 to 1474.7)	29.7 (27.5 to 31.6)	36.7 (33.2 to 40.1)	28.2 (24.4 to 33.4)	4.0 (3.4 to 4.7)
Angola	216.6 (183.0 to 260.2)	2138.0 (1806.0 to 2568.6)	9.4 (7.7 to 11.0)	60.0 (49.5 to 69.0)	25.6 (16.2 to 37.0)	9.4 (7.3 to 11.5)
Bangladesh	235.5 (202.4 to 279.9)	1215.7 (1045.0 to 1445.2)	53.9 (45.1 to 62.3)	25.2 (19.2 to 32.2)	20.5 (10.9 to 32.4)	6.7 (4.2 to 9.3)
Brazil	180.6 (125.5 to 256.9)	2197.5 (1527.4 to 3127.0)	0.1 (0.0 to 0.1)	91.5 (83.8 to 96.3)	7.2 (2.8 to 14.2)	0.7 (-2.4 to 3.7)
Cambodia	122.5 (109.6 to 137.8)	3142.7 (2812.2 to 3536.9)	28.9 (25.6 to 32.2)	67.8 (63.8 to 71.4)	3.3 (1.3 to 6.3)	3.8 (2.8 to 4.8)
Central African Republic	22.0 (20.7 to 23.7)	632.6 (593.8 to 682.2)	65.2 (60.4 to 69.4)	12.9 (11.0 to 14.8)	21.3 (16.4 to 27.0)	4.5 (3.2 to 5.7)
China	2037.3 (1490.6 to 2755.3)	2372.1 (1735.6 to 3208.1)	0.6 (0.5 to 0.8)	79.3 (65.6 to 89.5)	17.0 (7.7 to 30.9)	-1.3 (-4.4 to 1.9)
Congo	28.1 (23.1 to 34.2)	1408.3 (1157.7 to 1716.3)	26.9 (21.9 to 32.4)	45.3 (35.6 to 55.1)	26.6 (15.6 to 39.1)	4.9 (2.8 to 7.1)
Democratic People's Republic of Korea	41.3 (33.4 to 49.9)	871.1 (704.8 to 1052.8)	0.0 (0.0 to 0.0)	95.5 (91.1 to 98.0)	4.4 (1.9 to 8.8)	1.9 (0.2 to 3.6)
Democratic Republic of the Congo	112.5 (90.8 to 139.3)	287.0 (231.7 to 355.4)	30.5 (24.3 to 37.3)	12.7 (8.4 to 17.6)	52.5 (41.9 to 62.2)	3.0 (1.3 to 4.8)
Ethiopia	333.9 (284.1 to 400.1)	1453.2 (1236.4 to 1741.0)	23.6 (19.5 to 27.5)	41.5 (33.9 to 49.5)	24.0 (14.8 to 35.9)	2.5 (1.3 to 3.8)

India	7182.5 (5453.9 to 9512.0)	2470.9 (1876.3 to 3272.4)	5.9 (4.3 to 7.6)	47.7 (33.7 to 61.2)	43.8 (28.9 to 59.6)	7.9 (4.9 to 11.0)
Indonesia	608.8 (487.8 to 759.9)	1084.5 (868.9 to 1353.6)	26.4 (20.9 to 32.5)	56.8 (45.8 to 66.9)	14.5 (6.4 to 26.8)	2.6 (0.3 to 4.8)
Kenya	155.4 (133.7 to 183.9)	845.0 (727.4 to 1000.2)	44.1 (37.0 to 50.9)	33.5 (25.9 to 41.3)	14.7 (7.5 to 25.1)	5.5 (2.8 to 8.0)
Lesotho	61.2 (58.1 to 64.5)	2347.6 (2229.5 to 2475.3)	70.1 (66.4 to 73.7)	12.2 (9.0 to 15.7)	17.7 (14.7 to 21.0)	12.8 (11.1 to 14.4)
Liberia	5.8 (4.6 to 7.5)	518.5 (414.1 to 669.6)	33.1 (25.2 to 40.8)	33.1 (23.6 to 43.3)	30.9 (16.9 to 46.6)	5.0 (2.3 to 7.7)
Mozambique	176.8 (150.9 to 207.6)	784.7 (669.4 to 921.2)	40.1 (33.9 to 46.6)	27.6 (21.8 to 33.6)	30.7 (20.1 to 41.0)	8.3 (7.0 to 9.5)
Myanmar	285.3 (244.3 to 353.6)	2623.4 (2246.8 to 3251.5)	58.5 (46.8 to 67.7)	15.8 (10.8 to 21.2)	25.7 (14.8 to 40.5)	9.5 (7.5 to 11.6)
Namibia	176.2 (155.2 to 198.2)	9176.0 (8082.6 to 10323.5)	13.3 (11.8 to 15.0)	75.7 (71.5 to 79.5)	2.2 (1.1 to 3.8)	12.4 (10.2 to 14.3)
Nigeria	720.0 (504.3 to 1069.7)	1223.2 (856.7 to 1817.3)	27.7 (17.9 to 38.0)	19.3 (11.6 to 28.1)	51.6 (35.1 to 68.9)	5.6 (1.9 to 9.8)
Pakistan	786.2 (599.9 to 1067.4)	2134.1 (1628.5 to 2897.5)	38.5 (27.7 to 49.4)	15.1 (9.6 to 21.4)	43.8 (28.5 to 58.7)	-0.6 (-2.8 to 1.8)
Papua New Guinea	28.5 (24.8 to 33.2)	1894.6 (1647.0 to 2205.0)	40.2 (34.4 to 46.0)	57.5 (51.3 to 63.9)	2.3 (1.1 to 4.1)	15.4 (13.1 to 17.8)
Philippines	603.2 (496.0 to 721.5)	2542.8 (2090.9 to 3041.2)	22.5 (18.7 to 27.1)	72.9 (67.0 to 77.8)	4.1 (1.8 to 7.8)	7.4 (5.4 to 9.4)
Russian Federation	5740.4 (4956.5 to 6625.4)	52097.7 (44982.9 to 60128.8)	0.0 (0.0 to 0.0)	90.0 (83.3 to 94.8)	9.2 (4.4 to 16.0)	4.9 (3.4 to 6.4)
Sierra Leone	132.8 (119.4 to 146.8)	5620.4 (5052.4 to 6213.2)	21.5 (19.4 to 23.9)	71.6 (67.4 to 75.0)	3.4 (1.6 to 6.7)	9.1 (7.6 to 10.7)
South Africa	908.2 (724.5 to 1138.2)	2097.2 (1673.0 to 2628.3)	25.9 (20.4 to 32.0)	67.3 (59.3 to 74.4)	4.0 (1.7 to 7.5)	1.4 (-0.4 to 3.3)
Thailand	102.4 (79.5 to 131.1)	1753.1 (1360.8 to 2244.0)	23.3 (17.9 to 29.5)	64.5 (53.5 to 73.9)	10.7 (4.5 to 21.3)	1.3 (-1.1 to 3.8)
United Republic of Tanzania	310.8 (272.0 to 359.0)	1458.1 (1276.4 to 1684.6)	32.3 (27.8 to 36.7)	20.8 (15.5 to 27.2)	38.9 (31.5 to 46.7)	6.1 (4.6 to 7.7)
Viet Nam	206.1 (164.3 to 262.9)	1605.4 (1279.9 to 2047.6)	23.1 (17.9 to 28.6)	60.1 (48.1 to 69.8)	16.1 (7.4 to 29.1)	4.0 (2.0 to 6.0)
Zambia	270.7 (257.1 to 287.3)	2382.5 (2263.2 to 2529.1)	56.3 (53.0 to 59.3)	12.2 (9.0 to 16.1)	15.9 (12.9 to 19.0)	0.8 (0.3 to 1.3)
Zimbabwe	152.7 (134.3 to 175.9)	1145.7 (1007.8 to 1319.3)	34.1 (29.4 to 38.5)	19.6 (14.8 to 25.7)	37.0 (29.6 to 45.4)	3.9 (1.7 to 5.8)
Afghanistan	118.3 (102.7 to 143.8)	2882.5 (2501.9 to 3503.6)	58.5 (47.7 to 66.9)	19.6 (14.6 to 24.8)	21.9 (11.0 to 36.2)	9.6 (6.3 to 12.9)
Albania	10.1 (7.2 to 14.1)	23009.2 (16588.0 to 32182.1)	0.9 (0.6 to 1.3)	98.7 (98.1 to 99.2)	0.3 (0.1 to 0.7)	8.7 (5.4 to 11.8)
Algeria	121.0 (85.9 to 167.7)	8066.9 (5726.2 to 11176.8)	0.0 (0.0 to 0.0)	99.8 (99.6 to 99.9)	0.2 (0.1 to 0.4)	7.2 (4.3 to 10.1)
American Samoa	0.1 (0.1 to 0.1)	8940.0 (6081.1 to 12634.6)	0.0 (0.0 to 0.0)	98.6 (97.3 to 99.4)	1.3 (0.5 to 2.6)	6.4 (3.2 to 9.6)
Argentina	160.6 (111.3 to 233.9)	15940.9 (11041.4 to 23215.6)	0.0 (0.0 to 0.0)	99.8 (99.7 to 99.9)	0.1 (0.0 to 0.2)	3.4 (0.4 to 6.4)
Armenia	23.9 (19.5 to 28.6)	21344.8 (17468.4 to 25524.8)	20.1 (16.6 to 24.3)	76.2 (70.5 to 80.6)	3.7 (1.6 to 7.3)	9.6 (7.6 to 11.7)
Azerbaijan	152.5 (118.6 to 198.2)	13312.3 (10352.0 to 17309.1)	20.5 (15.5 to 25.9)	46.8 (34.0 to 58.2)	32.5 (18.4 to 49.6)	10.9 (8.1 to 13.9)
Belarus	337.9 (273.0 to 412.5)	91593.4 (73980.5 to 111796.5)	2.9 (2.4 to 3.6)	97.0 (96.3 to 97.6)	0.1 (0.0 to 0.1)	2.8 (1.1 to 4.5)
Belize	0.5 (0.5 to 0.6)	3985.1 (3400.8 to 4751.3)	52.6 (43.8 to 61.1)	43.7 (34.5 to 53.0)	3.4 (1.5 to 6.5)	7.4 (5.0 to 9.6)
Benin	36.0 (27.8 to 47.2)	1661.9 (1285.0 to 2178.6)	25.9 (19.5 to 33.0)	27.3 (19.3 to 36.1)	46.4 (33.3 to 60.0)	3.6 (1.2 to 6.2)
Bhutan	7.2 (5.5 to 9.0)	6745.7 (5210.0 to 8521.2)	25.5 (19.8 to 32.4)	74.2 (67.2 to 79.9)	0.3 (0.1 to 0.6)	5.5 (2.9 to 8.1)

Bolivia (Plurinational State of)	40.9 (33.0 to 51.0)	4246.6 (3429.8 to 5299.4)	29.6 (23.4 to 36.2)	56.0 (46.0 to 66.1)	13.5 (6.4 to 23.3)	2.7 (0.7 to 4.5)
Bosnia and Herzegovina	32.9 (23.2 to 44.4)	27535.3 (19439.8 to 37188.7)	0.1 (0.1 to 0.2)	99.1 (98.3 to 99.6)	0.8 (0.3 to 1.6)	6.3 (3.4 to 9.4)
Botswana	163.9 (142.1 to 189.4)	10308.8 (8934.7 to 11910.6)	6.9 (6.0 to 7.9)	76.2 (72.3 to 79.8)	3.3 (2.3 to 4.5)	5.4 (4.3 to 6.6)
Bulgaria	57.3 (41.4 to 78.4)	38242.3 (27632.3 to 52324.6)	6.6 (4.7 to 8.9)	91.9 (88.6 to 94.4)	1.5 (0.6 to 3.0)	0.2 (-2.2 to 2.8)
Burkina Faso	54.0 (41.2 to 71.8)	1033.4 (788.4 to 1372.2)	15.3 (11.3 to 19.6)	31.7 (22.2 to 42.1)	42.0 (27.6 to 56.1)	3.7 (1.7 to 5.6)
Burundi	67.6 (63.3 to 72.4)	1020.1 (954.5 to 1091.7)	32.9 (30.7 to 35.1)	23.2 (20.7 to 26.0)	32.8 (28.8 to 36.9)	3.8 (3.1 to 4.5)
Cabo Verde	2.5 (2.1 to 3.0)	3555.6 (3005.5 to 4200.4)	41.5 (34.9 to 48.8)	44.5 (35.7 to 54.0)	13.0 (6.5 to 22.5)	5.2 (3.2 to 7.3)
Cameroon	104.0 (65.4 to 160.8)	1468.5 (922.7 to 2270.4)	9.3 (5.7 to 14.1)	13.9 (7.6 to 21.4)	72.3 (58.6 to 83.6)	3.6 (-0.3 to 7.5)
Chad	67.4 (53.2 to 85.9)	1566.6 (1236.8 to 1997.1)	8.7 (6.7 to 10.8)	32.1 (24.5 to 41.0)	57.2 (46.0 to 66.9)	2.6 (0.6 to 4.5)
Colombia	182.3 (128.3 to 255.7)	15868.9 (11169.3 to 22261.2)	0.4 (0.3 to 0.5)	97.1 (94.7 to 98.6)	1.5 (0.6 to 3.3)	1.5 (-1.3 to 4.3)
Comoros	2.0 (1.4 to 2.8)	1205.7 (873.7 to 1748.8)	37.0 (24.8 to 49.6)	6.5 (4.0 to 9.9)	54.5 (39.0 to 69.8)	3.9 (-0.1 to 7.7)
Costa Rica	15.7 (10.6 to 21.7)	34423.2 (23342.4 to 47738.2)	0.0 (0.0 to 0.0)	99.8 (99.7 to 99.9)	0.2 (0.1 to 0.3)	10.1 (6.8 to 13.6)
Cuba	76.2 (53.4 to 108.5)	99204.2 (69560.9 to 141308.9)	0.0 (0.0 to 0.0)	99.7 (99.5 to 99.9)	0.2 (0.1 to 0.5)	6.1 (3.0 to 9.3)
Côte d'Ivoire	69.5 (55.1 to 91.1)	1101.6 (874.5 to 1443.9)	28.4 (21.3 to 35.2)	36.1 (25.8 to 46.8)	30.8 (16.5 to 46.7)	-0.6 (-3.3 to 2.0)
Djibouti	6.2 (5.4 to 7.1)	1504.5 (1312.0 to 1735.9)	54.2 (46.8 to 61.9)	33.7 (25.5 to 42.4)	11.8 (6.5 to 18.8)	6.6 (4.9 to 8.3)
Dominica	0.2 (0.1 to 0.2)	7589.8 (5845.5 to 10162.3)	18.6 (13.7 to 23.7)	76.8 (69.4 to 82.9)	4.4 (1.9 to 8.8)	1.4 (-1.1 to 4.2)
Dominican Republic	55.1 (40.5 to 75.3)	8883.0 (6522.1 to 12140.9)	5.7 (4.1 to 7.6)	91.8 (88.5 to 94.5)	1.9 (0.8 to 3.8)	6.5 (3.8 to 9.3)
Ecuador	29.6 (20.9 to 41.9)	5273.0 (3734.0 to 7472.1)	0.1 (0.0 to 0.1)	95.3 (90.8 to 98.1)	4.1 (1.5 to 8.8)	2.1 (-0.8 to 4.8)
Egypt	91.9 (66.8 to 123.5)	4941.8 (3589.1 to 6639.7)	0.2 (0.2 to 0.3)	80.4 (65.3 to 90.1)	17.4 (8.0 to 31.7)	3.9 (1.2 to 6.6)
El Salvador	16.4 (13.5 to 20.3)	9414.9 (7727.2 to 11624.3)	40.6 (32.5 to 48.9)	58.7 (50.3 to 66.8)	0.7 (0.3 to 1.4)	7.7 (5.4 to 10.1)
Equatorial Guinea	26.6 (18.7 to 38.1)	5637.9 (3962.9 to 8098.3)	0.2 (0.1 to 0.2)	43.2 (28.2 to 59.4)	55.8 (39.4 to 71.1)	8.4 (4.5 to 12.0)
Eritrea	17.6 (14.9 to 20.4)	605.5 (514.5 to 703.4)	9.5 (8.2 to 11.1)	10.4 (7.1 to 14.7)	76.9 (71.5 to 81.1)	1.7 (0.3 to 3.2)
Eswatini	49.4 (45.5 to 54.4)	4520.0 (4158.0 to 4973.2)	41.7 (37.8 to 45.2)	22.7 (17.6 to 29.2)	27.4 (23.7 to 31.0)	14.5 (12.2 to 16.6)
Fiji	7.0 (5.5 to 8.8)	20864.7 (16478.5 to 26255.1)	18.4 (14.5 to 23.0)	81.4 (76.8 to 85.4)	0.1 (0.0 to 0.2)	5.5 (3.3 to 7.7)
Gabon	10.0 (7.4 to 13.1)	1715.6 (1267.1 to 2251.5)	6.4 (4.7 to 8.4)	84.8 (77.1 to 90.0)	6.3 (2.7 to 12.9)	-1.9 (-4.2 to 0.5)
Gambia	22.9 (22.0 to 24.2)	3959.0 (3791.3 to 4175.4)	73.7 (69.8 to 76.9)	16.5 (14.2 to 18.9)	7.9 (4.6 to 12.2)	9.0 (7.9 to 10.0)
Georgia	65.0 (50.3 to 83.2)	23867.8 (18490.6 to 30583.5)	24.7 (19.0 to 31.4)	71.7 (64.4 to 78.6)	3.1 (1.4 to 6.2)	5.7 (3.2 to 8.3)
Ghana	98.8 (79.0 to 128.9)	1163.8 (930.9 to 1518.9)	37.7 (28.4 to 46.4)	24.6 (16.8 to 32.6)	34.3 (21.0 to 50.0)	7.1 (3.5 to 10.7)
Grenada	0.2 (0.1 to 0.2)	13881.3 (10612.4 to 18003.9)	18.2 (13.8 to 23.4)	76.8 (69.5 to 82.6)	4.8 (2.0 to 9.4)	-3.2 (-5.5 to -0.6)
Guatemala	15.1 (10.8 to 20.4)	4359.8 (3100.7 to 5878.5)	4.8 (3.5 to 6.6)	87.2 (79.7 to 92.3)	7.4 (3.1 to 14.8)	1.3 (-1.4 to 4.1)

Guinea	22.2 (17.5 to 29.5)	681.8 (538.3 to 905.1)	36.8 (27.2 to 45.8)	17.5 (11.7 to 23.9)	40.5 (25.6 to 56.2)	3.8 (0.8 to 7.0)
Guinea-Bissau	12.4 (10.8 to 14.7)	2569.2 (2250.0 to 3044.4)	54.4 (45.6 to 61.7)	20.9 (16.8 to 24.9)	23.7 (14.4 to 35.6)	7.1 (4.9 to 9.4)
Guyana	1.8 (1.4 to 2.4)	3858.6 (2971.7 to 5014.4)	24.8 (18.7 to 31.6)	74.8 (68.0 to 81.0)	0.4 (0.2 to 0.8)	5.6 (2.4 to 8.7)
Haiti	4.2 (3.2 to 5.3)	447.6 (347.6 to 573.0)	11.9 (9.1 to 15.1)	86.6 (82.8 to 89.8)	1.5 (0.6 to 2.9)	4.6 (2.2 to 7.0)
Honduras	13.1 (10.4 to 16.3)	3983.8 (3182.2 to 4970.1)	33.2 (26.3 to 41.1)	61.6 (52.1 to 69.8)	4.9 (2.0 to 9.6)	3.4 (1.3 to 5.6)
Iran (Islamic Republic of)	241.4 (168.0 to 343.9)	18634.9 (12968.2 to 26549.3)	0.0 (0.0 to 0.0)	99.4 (98.7 to 99.7)	0.6 (0.2 to 1.3)	9.2 (6.1 to 12.2)
Iraq	100.7 (77.1 to 134.0)	6048.8 (4635.6 to 8051.2)	7.8 (5.8 to 10.0)	78.0 (67.0 to 86.0)	14.1 (6.6 to 25.7)	7.0 (4.1 to 9.7)
Jamaica	8.4 (6.1 to 11.3)	17756.3 (12784.8 to 23799.0)	0.0 (0.0 to 0.0)	86.9 (78.2 to 93.0)	9.3 (4.0 to 17.7)	-1.0 (-3.5 to 1.6)
Jordan	7.8 (5.8 to 10.2)	7264.2 (5448.5 to 9477.0)	16.4 (12.3 to 21.5)	65.8 (53.7 to 75.6)	14.2 (6.3 to 26.4)	0.6 (-1.8 to 3.2)
Kazakhstan	1186.6 (953.9 to 1467.5)	85498.1 (68731.7 to 105742.4)	2.5 (2.0 to 3.1)	97.1 (96.3 to 97.7)	0.1 (0.0 to 0.1)	4.1 (2.6 to 5.7)
Kiribati	1.3 (1.1 to 1.6)	4239.3 (3496.3 to 5081.1)	30.6 (25.3 to 36.8)	69.2 (63.0 to 74.6)	0.2 (0.1 to 0.3)	4.1 (2.3 to 6.0)
Kyrgyzstan	160.0 (143.6 to 177.7)	22552.5 (20243.0 to 25059.2)	22.3 (20.0 to 24.7)	67.4 (62.2 to 71.5)	10.3 (6.4 to 15.9)	8.1 (7.0 to 9.3)
Lao People's Democratic Republic	20.1 (16.4 to 24.5)	2026.2 (1656.6 to 2473.6)	25.8 (20.9 to 31.3)	59.5 (50.4 to 67.9)	13.7 (6.8 to 23.9)	2.7 (0.6 to 4.7)
Lebanon	12.3 (9.0 to 16.5)	7546.8 (5543.9 to 10127.4)	7.6 (5.5 to 10.0)	79.9 (70.3 to 86.9)	8.9 (3.9 to 17.4)	-0.1 (-2.8 to 2.6)
Libya	14.8 (10.3 to 20.6)	7680.4 (5365.9 to 10705.7)	0.0 (0.0 to 0.0)	99.2 (98.4 to 99.6)	0.6 (0.2 to 1.3)	0.4 (-2.5 to 3.4)
Madagascar	25.7 (20.3 to 32.7)	521.4 (412.9 to 663.1)	17.4 (13.5 to 21.6)	44.3 (32.7 to 56.4)	25.7 (14.2 to 40.0)	3.3 (1.0 to 5.7)
Malawi	95.0 (88.9 to 102.0)	1006.0 (941.3 to 1080.8)	65.2 (60.6 to 69.6)	9.1 (6.4 to 12.5)	24.2 (19.8 to 28.8)	6.8 (6.2 to 7.5)
Malaysia	257.8 (186.4 to 345.2)	13952.4 (10087.8 to 18680.6)	0.0 (0.0 to 0.0)	98.9 (97.8 to 99.5)	0.9 (0.4 to 1.9)	7.8 (5.1 to 10.7)
Maldives	1.9 (1.3 to 2.7)	10288.7 (7077.2 to 14457.8)	0.0 (0.0 to 0.0)	98.2 (96.6 to 99.3)	1.3 (0.5 to 2.8)	5.5 (2.6 to 8.4)
Mali	37.4 (27.8 to 51.4)	1329.3 (989.5 to 1828.6)	0.4 (0.3 to 0.5)	39.3 (25.9 to 52.8)	56.4 (42.0 to 70.4)	4.9 (1.5 to 8.5)
Marshall Islands	0.8 (0.6 to 1.1)	8111.9 (5830.1 to 10828.1)	4.3 (3.1 to 5.8)	95.1 (93.3 to 96.5)	0.5 (0.2 to 1.0)	5.2 (2.5 to 7.9)
Mauritania	7.6 (5.9 to 10.3)	1752.6 (1349.5 to 2375.7)	28.6 (20.7 to 36.5)	31.7 (21.3 to 43.2)	37.7 (23.4 to 54.8)	8.5 (4.5 to 12.2)
Mauritius	2.7 (2.0 to 3.7)	10827.9 (7883.6 to 14618.5)	8.2 (5.9 to 10.9)	86.9 (80.8 to 91.5)	4.9 (1.9 to 9.8)	5.1 (2.5 to 7.8)
Mexico	118.7 (78.5 to 163.7)	5448.6 (3602.3 to 7513.6)	0.8 (0.6 to 1.2)	99.0 (98.5 to 99.3)	0.1 (0.0 to 0.1)	2.5 (-0.4 to 5.4)
Micronesia (Federated States of)	0.0 (0.0 to 0.0)	307.6 (261.0 to 364.8)	53.4 (44.6 to 62.4)	45.9 (36.8 to 55.0)	0.8 (0.3 to 1.6)	5.4 (2.9 to 7.8)
Mongolia	57.3 (48.3 to 68.2)	11281.5 (9503.0 to 13419.8)	20.1 (16.7 to 23.6)	72.5 (66.8 to 77.7)	7.1 (3.7 to 12.0)	8.2 (6.3 to 10.3)
Montenegro	2.6 (1.8 to 3.5)	26025.5 (17951.6 to 36077.0)	0.4 (0.3 to 0.5)	98.9 (98.1 to 99.4)	0.7 (0.3 to 1.4)	-0.3 (-2.9 to 2.4)
Morocco	89.8 (66.3 to 119.6)	1760.2 (1298.8 to 2343.4)	3.8 (2.8 to 5.0)	78.0 (63.8 to 87.4)	17.6 (8.3 to 31.9)	3.5 (0.5 to 6.4)
Nepal	65.9 (51.3 to 84.7)	1359.7 (1058.6 to 1748.5)	2.1 (1.6 to 2.7)	70.5 (56.5 to 81.5)	25.3 (14.0 to 39.9)	3.9 (1.3 to 6.4)
Nicaragua	23.0 (18.6 to 28.6)	10870.6 (8817.6 to 13539.4)	36.4 (28.9 to 44.3)	63.1 (55.2 to 70.7)	0.5 (0.2 to 1.0)	13.8 (11.3 to 16.5)
Niger	13.7 (8.3 to 21.9)	345.4 (208.8 to 554.0)	7.9 (4.6 to 12.2)	20.0 (10.7 to 32.4)	70.7 (54.8 to 83.9)	3.5 (-0.9 to 8.2)

North Macedonia	5.5 (4.0 to 7.4)	9611.4 (7011.1 to 12983.7)	4.1 (2.9 to 5.4)	83.2 (72.9 to 89.9)	11.6 (5.2 to 21.7)	-0.8 (-3.3 to 1.8)
Palestine	0.9 (0.6 to 1.2)	1351.8 (953.7 to 1881.8)	0.0 (0.0 to 0.0)	92.8 (86.1 to 96.9)	4.5 (1.7 to 9.3)	5.6 (2.6 to 8.5)
Paraguay	28.1 (21.5 to 36.1)	8312.3 (6358.1 to 10700.6)	16.6 (12.7 to 21.3)	71.4 (61.0 to 79.6)	10.8 (4.8 to 20.6)	6.7 (4.4 to 9.3)
Peru	273.9 (200.0 to 363.9)	9688.2 (7074.7 to 12869.4)	1.9 (1.4 to 2.5)	95.5 (92.7 to 97.2)	2.4 (1.0 to 4.9)	4.6 (2.3 to 7.0)
Republic of Moldova	97.1 (83.8 to 111.6)	29818.3 (25743.9 to 34285.7)	17.6 (15.2 to 20.2)	79.0 (75.4 to 82.1)	2.4 (1.1 to 4.5)	10.4 (8.7 to 12.4)
Romania	236.6 (172.2 to 328.8)	18917.6 (13766.7 to 26286.9)	3.9 (2.8 to 5.3)	95.9 (94.5 to 97.1)	0.2 (0.1 to 0.4)	0.9 (-1.7 to 3.7)
Rwanda	63.9 (57.6 to 70.8)	1388.8 (1252.3 to 1539.6)	41.7 (37.6 to 46.2)	34.6 (28.8 to 40.8)	13.0 (9.6 to 17.3)	4.7 (3.9 to 5.6)
Saint Lucia	0.3 (0.2 to 0.4)	9497.0 (7063.8 to 12888.5)	11.5 (8.3 to 15.1)	83.3 (76.7 to 88.3)	4.8 (1.9 to 10.0)	1.9 (-0.7 to 4.8)
Saint Vincent and the Grenadines	0.2 (0.2 to 0.3)	7885.4 (5919.4 to 10240.9)	17.9 (13.5 to 23.4)	77.5 (70.4 to 83.2)	4.3 (1.9 to 8.8)	2.0 (-0.4 to 4.6)
Samoa	0.2 (0.1 to 0.2)	2387.5 (1775.9 to 3146.1)	14.2 (10.6 to 18.7)	78.2 (70.2 to 84.6)	5.0 (2.0 to 10.6)	2.0 (-0.6 to 4.6)
Sao Tome and Principe	1.4 (1.3 to 1.5)	6708.4 (6192.4 to 7356.6)	46.5 (42.3 to 50.2)	19.1 (14.0 to 25.2)	7.0 (3.1 to 13.3)	-6.7 (-8.7 to -4.1)
Senegal	28.9 (23.3 to 36.5)	954.9 (767.5 to 1203.0)	30.6 (24.0 to 37.6)	41.7 (31.5 to 52.5)	24.4 (12.1 to 40.0)	3.4 (0.7 to 6.0)
Serbia	43.5 (30.8 to 60.7)	19708.7 (13935.5 to 27463.9)	0.1 (0.0 to 0.1)	96.6 (93.2 to 98.6)	3.3 (1.3 to 6.6)	3.5 (0.8 to 6.1)
Solomon Islands	2.2 (2.0 to 2.4)	4718.0 (4233.4 to 5266.8)	59.9 (53.5 to 66.5)	39.8 (33.1 to 46.3)	0.3 (0.1 to 0.5)	8.5 (6.6 to 10.6)
Somalia	48.2 (45.8 to 50.8)	951.0 (904.2 to 1003.7)	63.6 (60.2 to 66.9)	7.8 (6.5 to 9.1)	27.9 (24.4 to 31.6)	8.5 (7.5 to 9.6)
South Sudan	135.6 (126.7 to 144.8)	4318.0 (4032.6 to 4610.1)	40.2 (37.6 to 43.0)	6.2 (5.0 to 7.8)	47.0 (43.3 to 50.6)	7.2 (5.6 to 9.1)
Sri Lanka	65.5 (46.1 to 90.8)	6529.3 (4588.5 to 9041.9)	9.2 (6.4 to 12.6)	88.5 (84.0 to 92.1)	2.1 (0.9 to 4.4)	-7.6 (-10.3 to -5.1)
Sudan	57.4 (44.6 to 75.8)	1593.8 (1237.2 to 2102.7)	41.7 (31.1 to 52.8)	24.2 (15.9 to 33.4)	32.6 (16.8 to 50.2)	5.7 (2.8 to 8.8)
Suriname	2.5 (2.3 to 2.8)	20912.5 (18893.1 to 23631.1)	69.5 (61.3 to 76.6)	30.1 (22.9 to 38.4)	0.2 (0.1 to 0.4)	8.1 (5.8 to 10.4)
Syrian Arab Republic	40.6 (31.6 to 51.1)	10603.7 (8269.2 to 13339.6)	23.2 (18.1 to 29.3)	59.8 (49.4 to 69.4)	16.1 (8.1 to 27.2)	-0.2 (-2.6 to 2.3)
Tajikistan	119.8 (106.8 to 137.3)	12191.3 (10860.5 to 13964.9)	55.7 (48.5 to 62.3)	30.4 (24.1 to 37.5)	13.8 (7.6 to 22.8)	10.6 (9.0 to 12.1)
Timor-Leste	8.5 (7.0 to 10.4)	4088.5 (3373.6 to 5004.7)	46.4 (37.6 to 55.7)	53.0 (43.6 to 62.0)	0.4 (0.2 to 0.9)	8.6 (6.6 to 10.7)
Togo	14.5 (11.2 to 19.7)	801.8 (617.6 to 1085.1)	36.0 (26.0 to 45.7)	18.6 (12.0 to 26.0)	40.1 (25.4 to 57.2)	4.9 (1.7 to 8.3)
Tonga	0.3 (0.2 to 0.3)	7366.4 (5610.1 to 9606.0)	9.5 (7.1 to 12.2)	68.3 (56.5 to 78.7)	21.5 (11.7 to 34.0)	4.1 (1.5 to 6.9)
Tunisia	6.3 (4.4 to 8.8)	1988.8 (1387.7 to 2765.7)	0.3 (0.2 to 0.5)	94.5 (89.3 to 97.4)	5.0 (2.0 to 10.0)	4.7 (1.6 to 7.7)
Turkey	212.6 (148.5 to 303.4)	9303.6 (6498.2 to 13275.8)	0.0 (0.0 to 0.0)	97.3 (94.5 to 98.8)	2.4 (1.0 to 5.2)	0.9 (-1.7 to 3.9)
Turkmenistan	41.7 (32.5 to 52.6)	10203.0 (7958.5 to 12866.6)	14.0 (10.9 to 17.7)	66.9 (53.0 to 76.7)	18.5 (9.2 to 32.3)	6.9 (4.7 to 9.1)
Uganda	181.8 (148.6 to 233.5)	893.0 (730.0 to 1147.3)	39.4 (30.2 to 47.5)	17.4 (12.4 to 23.4)	40.8 (28.2 to 54.1)	2.9 (1.2 to 4.8)
Ukraine	708.0 (578.7 to 874.8)	22770.7 (18612.0 to 28136.4)	6.5 (5.2 to 7.9)	88.6 (84.3 to 91.8)	4.8 (2.2 to 9.2)	4.1 (2.1 to 6.3)
Uzbekistan	630.5 (507.6 to 769.5)	32239.4 (25959.3 to 39349.1)	11.7 (9.5 to 14.4)	67.9 (57.2 to 76.3)	20.2 (12.0 to 31.0)	9.6 (7.5 to 11.7)
Vanuatu	0.5 (0.4 to 0.7)	2649.6 (1968.5 to 3545.6)	3.3 (2.4 to 4.3)	92.4 (88.7 to 95.0)	3.8 (1.7 to 6.8)	4.8 (2.2 to 7.6)

Venezuela (Bolivarian Republic of)	39.4 (28.0 to 53.5)	5396.9 (3833.0 to 7313.4)	0.0 (0.0 to 0.0)	89.5 (80.4 to 95.0)	5.6 (2.3 to 11.5)	-5.6 (-8.2 to -2.8)
Yemen	7.2 (4.9 to 10.9)	441.4 (299.5 to 668.9)	23.2 (14.7 to 32.8)	23.3 (13.6 to 35.1)	52.9 (34.6 to 70.8)	-3.1 (-6.1 to 0.1)

Note:

Notified treated cases were reported by the ministry of health from each country to WHO. Non-notified treated cases were estimated by incidence net notification.

Tuberculosis development assistance for health (DAH) for 135 low- and middle-income countries includes spending on two parts: 1. Administration and global projects and 2. Country projects. Except that, Tuberculosis DAH only includes spending on country-specific projects for country groups and individual countries.

All countries in high burden country grouping are listed alphabetically and all remaining countries are listed alphabetically.

S6. How these estimates might be used

We expect that a panel of estimated data regarding total TB spending, consisting of four sources, for each low- and middle-income country (LMIC) and each year, would be used by academics, policy makers, practitioners, and other stakeholders. All the estimates are available to the public in 2019 USD, PPP, and as percentage of GDP. We expect that the estimates with uncertainty intervals, available through Global Health Data Exchange (GHDx) for the public, will enable a wide range use. For policy makers, we also have an interactive website for visualisation and use cases by policy makers and practitioners. In addition, we put all scientific evidence into lay language in our policy report “Financing Global Health,” for the broader audience who may or may not have academic interest to specify the underlying methods.

References for appendix

1 Jacquet V, Morose W, Schwartzman K, *et al.* Impact of DOTS expansion on tuberculosis related outcomes and costs in Haiti. *BMC Public Health* 2006; **6**: 209.

2 Collins D, Beyene D, Tedla Y, Diro E, Mesfin H, Levin A. Costs faced by multi-drug resistant tuberculosis patients during diagnosis and treatment. Report from a pilot study in Ethiopia. TB CARE I – Management Sciences for Health. Submitted to USAID by Management Sciences for Health for the TB CARE I Project., 2013 https://www.challengetb.org/publications/tools/costing/Costs_Faced_by_MDR-TB_Patients_During_Diagnosis_and_Treatment_Ethiopia.pdf (accessed May 9, 2019).

3 Abate KH, Abay SM, Abbafati C, *et al.* Global, regional, and national age-sex-specific mortality and life expectancy, 1950–2017: a systematic analysis for the Global Burden of Disease Study 2017. *The Lancet* 2018; **392**: 1684–735.

4 Global Burden of Disease Health Financing Collaborator Network. Health spending for Sustainable Development Goal 3: total, HIV/AIDS, tuberculosis, malaria, universal health service coverage, financial risk protection, and donor spending, 2000 to 2030 and 2050. 2019; published online Dec 13.

5 Ananthakrishnan R, Muniyandi M, Jeyaraj A, Palani G, Sathiyasekaran BWC. Expenditure Pattern for TB Treatment among Patients Registered in an Urban Government DOTS Program in Chennai City, South India. *Tuberc Res Treat* 2012; **2012**. DOI:10.1155/2012/747924.

6 Aspler A, Menzies D, Oxlade O, *et al.* Cost of tuberculosis diagnosis and treatment from the patient perspective in Lusaka, Zambia. *Int J Tuberc Lung Dis Off J Int Union Tuberc Lung Dis* 2008; **12**: 928–35.

7 Asres A, Jerene D, Deressa W. Pre- and post-diagnosis costs of tuberculosis to patients on Directly Observed Treatment Short course in districts of southwestern Ethiopia: a longitudinal study. *J Health Popul Nutr* 2018; **37**: 15.

8 Fuady A, Houweling TAJ, Mansyur M, Richardus JH. Catastrophic total costs in tuberculosis-affected households and their determinants since Indonesia's implementation of universal health coverage. *Infect Dis Poverty* 2018; **7**. DOI:10.1186/s40249-017-0382-3.

9 Jackson S, Sleigh AC, Wang GJ, Liu XL. Poverty and the economic effects of TB in rural China. *Int J Tuberc Lung Dis Off J Int Union Tuberc Lung Dis* 2006; **10**: 1104–10.

10 John KR, Daley P, Kincler N, Oxlade O, Menzies D. Costs incurred by patients with pulmonary tuberculosis in rural India. *Int J Tuberc Lung Dis Off J Int Union Tuberc Lung Dis* 2009; **13**: 1281–7.

11 Laokri S, Amoussouhui A, Ouendo EM, *et al.* A Care Pathway Analysis of Tuberculosis Patients in Benin: Highlights on Direct Costs and Critical Stages for an Evidence-Based Decision-Making. *PLoS ONE* 2014; **9**. DOI:10.1371/journal.pone.0096912.

12 Mahendradhata Y, Probandari A, Ahmad RA, *et al.* The Incremental Cost-Effectiveness of Engaging Private Practitioners to Refer Tuberculosis Suspects to DOTS Services in Jogjakarta, Indonesia. *Am J Trop Med Hyg* 2010; **82**: 1131–9.

13 Mauch V, Bonsu F, Gyapong M, *et al.* Free tuberculosis diagnosis and treatment are not enough: patient cost evidence from three continents. *Int J Tuberc Lung Dis Off J Int Union Tuberc Lung Dis* 2013; **17**: 381–7.

14 Othman GQ, Ibrahim MIM, Raja'a YA. Costs associated with tuberculosis diagnosis and treatment in Yemen for patients and public health services. *East Mediterr Health J Rev Sante Mediterr Orient Al-Majallah Al-Sihhiyah Li-Sharq Al-Mutawassit* 2012; **18**: 393–8.

15 Pantoja A, Floyd K, Unnikrishnan KP, *et al.* Economic evaluation of public-private mix for tuberculosis care and control, India. Part I. Socio-economic profile and costs among tuberculosis patients. *Int J Tuberc Lung Dis Off J Int Union Tuberc Lung Dis* 2009; **13**: 698–704.

16 The First Kenya Tuberculosis Patient Cost Survey 2017. The National Tuberculosis, Leprosy and Lung Disease Program https://www.chskenya.org/wp-content/uploads/2018/07/TB-Patient-Cost-Survey-2018.pdf (accessed May 10, 2019).

17 Pichenda K, Nakamura K, Morita A, Kizuki M, Seino K, Takano T. Non-hospital DOT and early diagnosis of tuberculosis reduce costs while achieving treatment success. *Int J Tuberc Lung Dis Off J Int Union Tuberc Lung Dis* 2012; **16**: 828–34.

18 Prasanna T, Jeyashree K, Chinnakali P, Bahurupi Y, Vasudevan K, Das M. Catastrophic costs of tuberculosis care: a mixed methods study from Puducherry, India. *Glob Health Action* 2018; **11**. DOI:10.1080/16549716.2018.1477493.

19 Rouzier VA, Oxlade O, Verduga R, Gresely L, Menzies D. Patient and family costs associated with tuberculosis, including multidrug-resistant tuberculosis, in Ecuador. *Int J Tuberc Lung Dis Off J Int Union Tuberc Lung Dis* 2010; **14**: 1316–22.

20 Vassall A, Chechulin Y, Raykhert I, *et al.* Reforming tuberculosis control in Ukraine: results of pilot projects and implications for the national scale-up of DOTS. *Health Policy Plan* 2009; **24**: 55–62.

21 Xu B, Dong HJ, Zhao Q, Bogg L. DOTS in China – removing barriers or moving barriers? *Health Policy Plan* 2006; **21**: 365–72.

22 WHO. Global Tuberculosis Report 2019. Geneva: World Health Organisation, 2019 https://apps.who.int/iris/bitstream/handle/10665/329368/9789241565714-eng.pdf?ua=1 (accessed Oct 25, 2019).

23 Stenberg K, Lauer JA, Gkountouras G, Fitzpatrick C, Stanciole A. Econometric estimation of WHO-CHOICE country-specific costs for inpatient and outpatient health service delivery. *Cost Eff Resour Alloc* 2018; **16**: 11.

24 Moses MW, Pedroza P, Baral R, *et al.* Funding and services needed to achieve universal health coverage: applications of global, regional, and national estimates of utilisation of outpatient visits and inpatient admissions from 1990 to 2016, and unit costs from 1995 to 2016. *Lancet Public Health* 2019; **4**: e49–73.
